# Supplementary material for: Cost-benefit tradeoff mediates the transition from rule-based to memory-based processing during practice
Source: PLoS Biol. 2025 Jan 23;23(1):e3002987. doi: 10.1371/journal.pbio.3002987 (PMC11793810; doi:10.1371/journal.pbio.3002987)
Supplement: S1 Table — (PDF) [file pbio.3002987.s001.pdf]

AllEffects

1.1. Replay effect in replay trial

HCPex region number	HCPex region name	p(one-tail)	FDR-p(one-tail)
1	Primary_Visual_Cortex_L	0.000252	0.003922
3	Third_Visual_Area_L	0.000280	0.004187
4	Fourth_Visual_Area_L	0.000310	0.004260
5	IntraParietal_Sulcus_Area_1_L	0.000439	0.004429
9	Area_V6A_L	0.000417	0.004379
12	Posterior_InferoTemporal_complex_L	0.000403	0.004350
15	VentroMedial_Visual_Area_2_L	0.000036	0.002940
19	Area_Lateral_Occipital_1_L	0.000244	0.003922
23	Middle_Temporal_Area_L	0.000119	0.003842
32	Area_23c_L	0.000246	0.003922
40	Supplementary_and_Cingulate_Eye_Field_L	0.000667	0.004758
45	Ventral_Area_6_L	0.000594	0.004758
64	Area_STSd_posterior_L	0.000160	0.003922
73	Area_Frontal_Opercular_5_L	0.000335	0.004260
75	Middle_Insular_Area_L	0.000167	0.003922
76	Para-Insular_Area_L	0.000666	0.004758
79	Posterior_Insular_Area_2_L	0.000656	0.004758
85	ParaHippocampal_Area_1_L	0.000229	0.003922
88	Area_PHT_L	0.000733	0.004916
89	Area_TE1_anterior_L	0.000362	0.004260
92	Area_TE2_anterior_L	0.000086	0.003402
98	Area_TemporoParietoOccipital_Junction_1_L	0.000691	0.004758
102	Medial_Area_7A_L	0.000576	0.004758
107	Area_Lateral_IntraParietal_dorsal_L	0.000141	0.003922
108	Area_Lateral_IntraParietal_ventral_L	0.000313	0.004260
110	Ventral_IntraParietal_Complex_L	0.000210	0.003922
111	Area_IntraParietal_0_L	0.000042	0.002940
119	Area_PGp_L	0.000936	0.005873
127	Dorsal_Transitional_Visual_Area_L	0.000891	0.005685
128	PreCuneus_Visual_Area_L	0.000095	0.003402
138	Area_8BM_L	0.000670	0.004758
142	Area_anterior_32_prime_L	0.000088	0.003402
143	Area_dorsal_32_L	0.000602	0.004758
144	Area_posterior_24_L	0.000880	0.005685
159	Area_44_L	0.000974	0.006012
165	Area_IFSa_L	0.000096	0.003402
166	Area_IFSp_L	0.000658	0.004758
172	Area_8C_L	0.000607	0.004758
173	Area_9-46d_L	0.000332	0.004260
175	Area_9_Posterior_L	0.000006	0.001596
176	Area_anterior_9-46v_L	0.000686	0.004758
181	Primary_Visual_Cortex_R	0.000153	0.003922
184	Fourth_Visual_Area_R	0.000677	0.004758

191	Fusiform_Face_Complex_R	0.000485	0.004492
195	VentroMedial_Visual_Area_2_R	0.000008	0.001596
197	Ventral_Visual_Complex_R	0.000383	0.004260
205	Area_V3CD_R	0.000614	0.004758
219	Area_6mp_R	0.000781	0.005151
222	Area_6_anterior_R	0.000371	0.004260
227	Premotor_Eye_Field_R	0.000473	0.004492
249	Anterior_Ventral_Insular_Area_R	0.000483	0.004492
252	Frontal_Opercular_Area_4_R	0.000353	0.004260
264	Area_TF_R	0.000062	0.003402
267	ParaHippocampal_Area_3_R	0.000251	0.003922
273	Area_TE2_posterior_R	0.000198	0.003922
288	Area_Lateral_IntraParietal_ventral_R	0.000183	0.003922
299	Area_PGp_R	0.000697	0.004758
318	Area_8BM_R	0.000194	0.003922
340	Area_45_R	0.000382	0.004260
341	Area_47l_(47_lateral)_R	0.000444	0.004429
344	Area_IFJp_R	0.000591	0.004758
353	Area_9-46d_R	0.000023	0.002940
382	Putamen_L	0.000045	0.002940

1.2. Replay effect in memory trial

HCPex region number	HCPex region name	p(one-tail)	FDR-p(one-tail)
Null	Null	Null	Null

1.3. Higher Replay effect in replay than memory trials

HCPex region number	HCPex region name	p(one-tail)	FDR-p(one-tail)
1	Primary_Visual_Cortex_L	0.000277	0.002532
2	Second_Visual_Area_L	0.000507	0.003170
3	Third_Visual_Area_L	0.000033	0.001263
4	Fourth_Visual_Area_L	0.000034	0.001263
5	IntraParietal_Sulcus_Area_1_L	0.000094	0.001500
11	Fusiform_Face_Complex_L	0.000074	0.001361
13	Eighth_Visual_Area_L	0.000498	0.003170
15	VentroMedial_Visual_Area_2_L	0.000003	0.000759
17	Ventral_Visual_Complex_L	0.000782	0.004343
24	Area_PH_L	0.000802	0.004393
26	Area_V4t_L	0.000898	0.004424
31	Primary_Motor_Cortex_L	0.000084	0.001428
35	Area_5L_L	0.000396	0.002854
40	Supplementary_and_Cingulate_Eye_Field_L	0.000467	0.003170
42	Area_6_anterior_L	0.000885	0.004424
43	Dorsal_area_6_L	0.000062	0.001263
44	Rostral_Area_6_L	0.000865	0.004424
45	Ventral_Area_6_L	0.000024	0.001263

46	Frontal_Eye_Fields_L	0.000048	0.001263
56	Medial_Belt_Complex_L	0.000195	0.002186
57	ParaBelt_Complex_L	0.000077	0.001361
60	Auditory_4_Complex_L	0.000391	0.002854
61	Auditory_5_Complex_L	0.000112	0.001561
67	Area_TA2_L	0.000321	0.002603
79	Posterior_Insular_Area_2_L	0.000017	0.001263
81	PreSubiculum_L	0.000224	0.002263
88	Area_PHT_L	0.000208	0.002186
89	Area_TE1_anterior_L	0.000030	0.001263
90	Area_TE1_Middle_L	0.000495	0.003170
92	Area_TE2_anterior_L	0.000053	0.001263
97	Superior_Temporal_Visual_Area_L	0.000133	0.001721
98	Area_TemporoParietoOccipital_Junction_1_L	0.000175	0.002130
102	Medial_Area_7A_L	0.000297	0.002532
107	Area_Lateral_IntraParietal_dorsal_L	0.000004	0.000759
108	Area_Lateral_IntraParietal_ventral_L	0.000137	0.001721
109	Medial_IntraParietal_Area_L	0.000200	0.002186
110	Ventral_IntraParietal_Complex_L	0.000035	0.001263
111	Area_IntraParietal_0_L	0.000066	0.001278
112	Area_IntraParietal_1_L	0.000942	0.004522
113	Area_IntraParietal_2_L	0.000513	0.003170
118	Area_PGi_L	0.000637	0.003756
119	Area_PGp_L	0.000206	0.002186
123	Area_31pd_L	0.000348	0.002704
128	PreCuneus_Visual_Area_L	0.000055	0.001263
130	Parieto-Occipital_Sulcus_Area_2_L	0.000057	0.001263
132	RetroSplenial_Complex_L	0.000227	0.002263
138	Area_8BM_L	0.000883	0.004424
142	Area_anterior_32_prime_L	0.000006	0.000785
147	Area_p32_prime_L	0.000667	0.003870
160	Area_45_L	0.000895	0.004424
165	Area_IFSa_L	0.000260	0.002531
166	Area_IFSp_L	0.000982	0.004601
172	Area_8C_L	0.000045	0.001263
175	Area_9_Posterior_L	0.000291	0.002532
177	Inferior_6-8_Transitional_Area_L	0.000343	0.002704
178	Area_posterior_9-46v_L	0.000293	0.002532
181	Primary_Visual_Cortex_R	0.000019	0.001263
184	Fourth_Visual_Area_R	0.000427	0.003023
191	Fusiform_Face_Complex_R	0.000118	0.001577
195	VentroMedial_Visual_Area_2_R	0.000047	0.001263
197	Ventral_Visual_Complex_R	0.000629	0.003756
199	Area_Lateral_Occipital_1_R	0.000686	0.003926
205	Area_V3CD_R	0.000480	0.003170
221	Area_55b_R	0.000941	0.004522
222	Area_6_anterior_R	0.000299	0.002532

226	Frontal_Eye_Fields_R	0.000834	0.004424
227	Premotor_Eye_Field_R	0.000501	0.003170
235	Lateral_Belt_Complex_R	0.000203	0.002186
236	Medial_Belt_Complex_R	0.000291	0.002532
252	Frontal_Opercular_Area_4_R	0.000959	0.004550
273	Area_TE2_posterior_R	0.000098	0.001500
278	Area_TemporoParietoOccipital_Junction_1_R	0.000863	0.004424
288	Area_Lateral_IntraParietal_ventral_R	0.000056	0.001263
309	Parieto-Occipital_Sulcus_Area_1_R	0.000697	0.003927
310	Parieto-Occipital_Sulcus_Area_2_R	0.000371	0.002821
335	Area_47s_R	0.000443	0.003077
350	Area_8Av_R	0.000104	0.001500
353	Area_9-46d_R	0.000616	0.003742
357	Inferior_6-8_Transitional_Area_R	0.000871	0.004424
358	Area_posterior_9-46v_R	0.000319	0.002603
382	Putamen_L	0.000060	0.001263
415	Putamen_R	0.000101	0.001500
416	Caudate_R	0.000377	0.002821

1.4. Cue effect in replay trial

HCPex region number	HCPex region name	p(one-tail)	FDR-p(one-tail)
1	Primary_Visual_Cortex_L	0.000826	0.021414
27	Area_1_L	0.000002	0.000294
28	Area_2_L	0.000458	0.017823
30	Primary_Sensory_Cortex_L	0.000049	0.003197
31	Primary_Motor_Cortex_L	0.000214	0.009267
33	Dorsal_Area_24d_L	0.000734	0.021414
43	Dorsal_area_6_L	0.000150	0.008158
52	Area_OP4-PV_L	0.000693	0.021414
207	Area_1_R	0.000002	0.000294
208	Area_2_R	0.000023	0.001819
209	Area_3a_R	0.000017	0.001650
210	Primary_Sensory_Cortex_R	0.000001	0.000294
211	Primary_Motor_Cortex_R	0.000782	0.021414
294	Area_PF_Complex_R	0.000546	0.019296
297	Area_PFt_R	0.000168	0.008158

1.5. Cue effect in memory trial

HCPex region number	HCPex region name	p(one-tail)	FDR-p(one-tail)
1	Primary_Visual_Cortex_L	0.000000	0.000010
2	Second_Visual_Area_L	0.000000	0.000005
3	Third_Visual_Area_L	0.000003	0.000047
4	Fourth_Visual_Area_L	0.000002	0.000035
5	IntraParietal_Sulcus_Area_1_L	0.000994	0.002930
11	Fusiform_Face_Complex_L	0.000070	0.000453

17	Ventral_Visual_Complex_L	0.000467	0.001818
24	Area_PH_L	0.000501	0.001857
27	Area_1_L	0.000001	0.000021
28	Area_2_L	0.000009	0.000100
29	Area_3a_L	0.000022	0.000201
30	Primary_Sensory_Cortex_L	0.000000	0.000002
31	Primary_Motor_Cortex_L	0.000000	0.000001
33	Dorsal_Area_24d_L	0.000000	0.000013
35	Area_5L_L	0.000075	0.000473
38	Area_6m_anterior_L	0.000807	0.002533
40	Supplementary_and_Cingulate_Eye_Field_L	0.000018	0.000166
41	Area_55b_L	0.000122	0.000680
42	Area_6_anterior_L	0.000077	0.000474
43	Dorsal_area_6_L	0.000013	0.000141
44	Rostral_Area_6_L	0.000484	0.001835
45	Ventral_Area_6_L	0.000033	0.000258
46	Frontal_Eye_Fields_L	0.000002	0.000038
48	Area_43_L	0.000008	0.000098
50	Area_OP1-SII_L	0.000036	0.000271
51	Area_OP2-3-VS_L	0.000387	0.001599
52	Area_OP4-PV_L	0.000362	0.001533
71	Frontal_Opercular_Area_3_L	0.000891	0.002675
74	Insular_Granular_Complex_L	0.000154	0.000810
78	Area_Posterior_Insular_1_L	0.000726	0.002355
88	Area_PHT_L	0.000025	0.000219
93	Area_TE2_posterior_L	0.000424	0.001683
98	Area_TemporoParietoOccipital_Junction_1_L	0.000200	0.000980
102	Medial_Area_7A_L	0.000099	0.000577
103	Area_7PC_L	0.000012	0.000135
106	Anterior_IntraParietal_Area_L	0.000064	0.000444
107	Area_Lateral_IntraParietal_dorsal_L	0.000333	0.001422
108	Area_Lateral_IntraParietal_ventral_L	0.000002	0.000035
110	Ventral_IntraParietal_Complex_L	0.000480	0.001835
111	Area_IntraParietal_0_L	0.000418	0.001675
112	Area_IntraParietal_1_L	0.000551	0.001996
113	Area_IntraParietal_2_L	0.000204	0.000980
114	Area_PF_Complex_L	0.000000	0.000000
115	Area_PFm_Complex_L	0.000001	0.000017
116	Area_PF_Opercular_L	0.000204	0.000980
117	Area_PFt_L	0.000112	0.000632
118	Area_PGi_L	0.000628	0.002186
119	Area_PGp_L	0.000004	0.000047
120	Area_PGs_L	0.000184	0.000929
121	Area_23d_L	0.000538	0.001974
122	Area_31a_L	0.000103	0.000588
123	Area_31pd_L	0.000037	0.000271
124	Area_31p_ventral_L	0.000131	0.000699

125	Area_7m_L	0.000010	0.000119
126	Area_dorsal_23_a+b_L	0.000641	0.002186
128	PreCuneus_Visual_Area_L	0.000032	0.000254
130	Parieto-Occipital_Sulcus_Area_2_L	0.000003	0.000046
138	Area_8BM_L	0.000894	0.002675
142	Area_anterior_32_prime_L	0.000554	0.001996
159	Area_44_L	0.000491	0.001837
160	Area_45_L	0.000014	0.000142
161	Area_47l_(47_lateral)_L	0.000640	0.002186
165	Area_IFSa_L	0.000079	0.000474
166	Area_IFSp_L	0.000661	0.002204
167	Area_posterior_47r_L	0.000880	0.002675
168	Area_46_L	0.000842	0.002600
172	Area_8C_L	0.000130	0.000699
177	Inferior_6-8_Transitional_Area_L	0.000067	0.000453
178	Area_posterior_9-46v_L	0.000382	0.001599
181	Primary_Visual_Cortex_R	0.000000	0.000000
182	Second_Visual_Area_R	0.000000	0.000002
183	Third_Visual_Area_R	0.000002	0.000039
184	Fourth_Visual_Area_R	0.000003	0.000042
185	IntraParietal_Sulcus_Area_1_R	0.000270	0.001223
188	Sixth_Visual_Area_R	0.000003	0.000042
190	Seventh_Visual_Area_R	0.000317	0.001368
192	Posterior_InferoTemporal_complex_R	0.000766	0.002441
193	Eighth_Visual_Area_R	0.000055	0.000390
196	VentroMedial_Visual_Area_3_R	0.000007	0.000087
198	Area_FST_R	0.000669	0.002204
199	Area_Lateral_Occipital_1_R	0.000078	0.000474
200	Area_Lateral_Occipital_2_R	0.000415	0.001675
201	Area_Lateral_Occipital_3_R	0.000664	0.002204
202	Medial_Superior_Temporal_Area_R	0.000919	0.002730
203	Middle_Temporal_Area_R	0.000662	0.002204
205	Area_V3CD_R	0.000236	0.001105
206	Area_V4t_R	0.000276	0.001236
207	Area_1_R	0.000000	0.000010
208	Area_2_R	0.000003	0.000047
209	Area_3a_R	0.000000	0.000003
210	Primary_Sensory_Cortex_R	0.000000	0.000002
211	Primary_Motor_Cortex_R	0.000000	0.000012
213	Dorsal_Area_24d_R	0.000000	0.000007
215	Area_5L_R	0.000173	0.000886
216	Area_5m_R	0.000635	0.002186
218	Area_6m_anterior_R	0.000256	0.001170
219	Area_6mp_R	0.000069	0.000453
223	Dorsal_area_6_R	0.000017	0.000162
225	Ventral_Area_6_R	0.000069	0.000453
226	Frontal_Eye_Fields_R	0.000001	0.000014

230	Area_OP1-SII_R	0.000000	0.000000
237	ParaBelt_Complex_R	0.000739	0.002377
258	Area_Posterior_Insular_1_R	0.000580	0.002070
266	ParaHippocampal_Area_2_R	0.000246	0.001138
278	Area_TemporoParietoOccipital_Junction_1_R	0.000089	0.000526
280	Area_TemporoParietoOccipital_Junction_3_R	0.000125	0.000688
281	Lateral_Area_7A_R	0.000435	0.001707
282	Medial_Area_7A_R	0.000310	0.001354
283	Area_7PC_R	0.000036	0.000271
284	Lateral_Area_7P_R	0.000027	0.000223
286	Anterior_IntraParietal_Area_R	0.000486	0.001835
287	Area_Lateral_IntraParietal_dorsal_R	0.000391	0.001599
288	Area_Lateral_IntraParietal_ventral_R	0.000045	0.000324
289	Medial_IntraParietal_Area_R	0.000002	0.000038
291	Area_IntraParietal_0_R	0.000214	0.001014
292	Area_IntraParietal_1_R	0.000202	0.000980
293	Area_IntraParietal_2_R	0.000003	0.000042
294	Area_PF_Complex_R	0.000026	0.000219
295	Area_PFm_Complex_R	0.000018	0.000169
298	Area_PGi_R	0.000791	0.002501
300	Area_PGs_R	0.000014	0.000142
304	Area_31p_ventral_R	0.000026	0.000219
305	Area_7m_R	0.000891	0.002675
306	Area_dorsal_23_a+b_R	0.000008	0.000098
310	Parieto-Occipital_Sulcus_Area_2_R	0.000703	0.002298
348	Area_46_R	0.000620	0.002186
350	Area_8Av_R	0.000013	0.000139
357	Inferior_6-8_Transitional_Area_R	0.000074	0.000470
358	Area_posterior_9-46v_R	0.000031	0.000252
381	Thal_Ventral_posterolateral_L	0.000163	0.000846
382	Putamen_L	0.000287	0.001268
415	Putamen_R	0.000831	0.002585

1.6. Higher Cue effect in memory than replay trials

HCPex region number	HCPex region name	p(one-tail)	FDR-p(one-tail)
2	Second_Visual_Area_L	0.000976	0.011167
3	Third_Visual_Area_L	0.000208	0.004042
4	Fourth_Visual_Area_L	0.000004	0.000782
11	Fusiform_Face_Complex_L	0.000571	0.007940
15	VentroMedial_Visual_Area_2_L	0.000010	0.000989
35	Area_5L_L	0.000420	0.006290
64	Area_STSd_posterior_L	0.000030	0.001218
81	PreSubiculum_L	0.000050	0.001385
85	ParaHippocampal_Area_1_L	0.000031	0.001218
88	Area_PHT_L	0.000479	0.006906
98	Area_TemporoParietoOccipital_Junction_1_L	0.000334	0.005197

107	Area_Lateral_IntraParietal_dorsal_L	0.000965	0.011167
108	Area_Lateral_IntraParietal_ventral_L	0.000003	0.000782
110	Ventral_IntraParietal_Complex_L	0.000028	0.001218
122	Area_31a_L	0.000286	0.004639
128	PreCuneus_Visual_Area_L	0.000029	0.001218
142	Area_anterior_32_prime_L	0.000977	0.011167
160	Area_45_L	0.000049	0.001385
165	Area_IFSa_L	0.000067	0.001727
166	Area_IFSp_L	0.000008	0.000989
181	Primary_Visual_Cortex_R	0.000040	0.001384
182	Second_Visual_Area_R	0.000043	0.001384
184	Fourth_Visual_Area_R	0.000271	0.004589
185	IntraParietal_Sulcus_Area_1_R	0.000882	0.011062
195	VentroMedial_Visual_Area_2_R	0.000017	0.001218
196	VentroMedial_Visual_Area_3_R	0.000262	0.004589
197	Ventral_Visual_Complex_R	0.000026	0.001218
199	Area_Lateral_Occipital_1_R	0.000222	0.004114
200	Area_Lateral_Occipital_2_R	0.000841	0.010900
284	Lateral_Area_7P_R	0.000596	0.007994
288	Area_Lateral_IntraParietal_ventral_R	0.000130	0.002965
289	Medial_IntraParietal_Area_R	0.000203	0.004042
291	Area_IntraParietal_0_R	0.000115	0.002808
382	Putamen_L	0.000187	0.004042

1.7. Double dissociation effect (Conjunction)

HCPex region number	HCPex region name	p(one-tail)	FDR-p(one-tail)
2	Second_Visual_Area_L	0.000029	0.000254
3	Third_Visual_Area_L	0.000001	0.000033
4	Fourth_Visual_Area_L	0.000001	0.000030
11	Fusiform_Face_Complex_L	0.000004	0.000081
15	VentroMedial_Visual_Area_2_L	0.000000	0.000007
35	Area_5L_L	0.000019	0.000185
81	PreSubiculum_L	0.000004	0.000085
88	Area_PHT_L	0.000009	0.000122
98	Area_TemporoParietoOccipital_Junction_1_L	0.000004	0.000081
107	Area_Lateral_IntraParietal_dorsal_L	0.000000	0.000022
108	Area_Lateral_IntraParietal_ventral_L	0.000002	0.000067
110	Ventral_IntraParietal_Complex_L	0.000001	0.000030
128	PreCuneus_Visual_Area_L	0.000001	0.000037
142	Area_anterior_32_prime_L	0.000000	0.000022
160	Area_45_L	0.000024	0.000214
165	Area_IFSa_L	0.000003	0.000073
166	Area_IFSp_L	0.000011	0.000139
181	Primary_Visual_Cortex_R	0.000000	0.000013
184	Fourth_Visual_Area_R	0.000017	0.000171
195	VentroMedial_Visual_Area_2_R	0.000001	0.000030

197	Ventral_Visual_Complex_R	0.000013	0.000153
199	Area_Lateral_Occipital_1_R	0.000033	0.000273
288	Area_Lateral_IntraParietal_ventral_R	0.000001	0.000039
382	Putamen_L	0.000001	0.000051

AllEffects: regress out influence of RT

2.1. Rule effect in rule trial

HCPex region number	HCPex region name	p(one-tail)	FDR-p(one-tail)
1	Primary_Visual_Cortex_L	0.000188	0.002107
2	Second_Visual_Area_L	0.000799	0.003573
3	Third_Visual_Area_L	0.000139	0.002082
4	Fourth_Visual_Area_L	0.000165	0.002107
5	IntraParietal_Sulcus_Area_1_L	0.000244	0.002229
9	Area_V6A_L	0.000180	0.002107
11	Fusiform_Face_Complex_L	0.000654	0.003347
12	Posterior_InferoTemporal_complex_L	0.000217	0.002229
13	Eighth_Visual_Area_L	0.000486	0.002868
15	VentroMedial_Visual_Area_2_L	0.000050	0.001755
19	Area_Lateral_Occipital_1_L	0.000183	0.002107
20	Area_Lateral_Occipital_2_L	0.000996	0.003620
23	Middle_Temporal_Area_L	0.000073	0.001991
25	Area_V3CD_L	0.000985	0.003614
26	Area_V4t_L	0.000920	0.003587
31	Primary_Motor_Cortex_L	0.000962	0.003587
32	Area_23c_L	0.000068	0.001991
37	Area_5m_ventral_L	0.000935	0.003587
38	Area_6m_anterior_L	0.000893	0.003587
39	Area_6mp_L	0.000875	0.003587
40	Supplementary_and_Cingulate_Eye_Field_L	0.000259	0.002244
44	Rostral_Area_6_L	0.000524	0.002914
45	Ventral_Area_6_L	0.000327	0.002411
60	Auditory_4_Complex_L	0.000569	0.003026
61	Auditory_5_Complex_L	0.000713	0.003447
64	Area_STSd_posterior_L	0.000087	0.001991
65	Area_STSv_anterior_L	0.000785	0.003573
66	Area_STSv_posterior_L	0.000879	0.003587
69	Anterior_Ventral_Insular_Area_L	0.000954	0.003587
73	Area_Frontal_Opercular_5_L	0.000124	0.002016
75	Middle_Insular_Area_L	0.000030	0.001640
76	Para-Insular_Area_L	0.000406	0.002632
79	Posterior_Insular_Area_2_L	0.000110	0.001991
85	ParaHippocampal_Area_1_L	0.000298	0.002275
88	Area_PHT_L	0.000239	0.002229
89	Area_TE1_anterior_L	0.000139	0.002082
92	Area_TE2_anterior_L	0.000087	0.001991

96	PeriSylvian_Language_Area_L	0.000589	0.003053
98	Area_TemporoParietoOccipital_Junction_1_L	0.000288	0.002275
100	Area_TemporoParietoOccipital_Junction_3_L	0.000929	0.003587
102	Medial_Area_7A_L	0.000195	0.002107
104	Lateral_Area_7P_L	0.000812	0.003573
105	Medial_Area_7P_L	0.000509	0.002914
107	Area_Lateral_IntraParietal_dorsal_L	0.000113	0.001991
108	Area_Lateral_IntraParietal_ventral_L	0.000194	0.002107
109	Medial_IntraParietal_Area_L	0.000962	0.003587
110	Ventral_IntraParietal_Complex_L	0.000104	0.001991
111	Area_IntraParietal_0_L	0.000035	0.001640
113	Area_IntraParietal_2_L	0.000894	0.003587
119	Area_PGp_L	0.000470	0.002868
127	Dorsal_Transitional_Visual_Area_L	0.000391	0.002604
128	PreCuneus_Visual_Area_L	0.000028	0.001640
130	Parieto-Occipital_Sulcus_Area_2_L	0.000576	0.003026
132	RetroSplenial_Complex_L	0.000753	0.003489
138	Area_8BM_L	0.000172	0.002107
142	Area_anterior_32_prime_L	0.000042	0.001640
143	Area_dorsal_32_L	0.000335	0.002411
144	Area_posterior_24_L	0.000520	0.002914
147	Area_p32_prime_L	0.000507	0.002914
159	Area_44_L	0.000293	0.002275
162	Area_anterior_47r_L	0.000675	0.003411
165	Area_IFSa_L	0.000023	0.001640
166	Area_IFSp_L	0.000283	0.002275
168	Area_46_L	0.000429	0.002738
172	Area_8C_L	0.000265	0.002244
173	Area_9-46d_L	0.000118	0.001991
175	Area_9_Posterior_L	0.000002	0.000971
176	Area_anterior_9-46v_L	0.000233	0.002229
181	Primary_Visual_Cortex_R	0.000092	0.001991
184	Fourth_Visual_Area_R	0.000395	0.002604
191	Fusiform_Face_Complex_R	0.000274	0.002269
195	VentroMedial_Visual_Area_2_R	0.000009	0.001212
197	Ventral_Visual_Complex_R	0.000333	0.002411
205	Area_V3CD_R	0.000472	0.002868
213	Dorsal_Area_24d_R	0.000487	0.002868
219	Area_6mp_R	0.000375	0.002604
222	Area_6_anterior_R	0.000246	0.002229
224	Rostral_Area_6_R	0.000704	0.003447
227	Premotor_Eye_Field_R	0.000239	0.002229
249	Anterior_Ventral_Insular_Area_R	0.000175	0.002107
252	Frontal_Opercular_Area_4_R	0.000170	0.002107
253	Area_Frontal_Opercular_5_R	0.000939	0.003587
264	Area_TF_R	0.000026	0.001640
267	ParaHippocampal_Area_3_R	0.000236	0.002229

273	Area_TE2_posterior_R	0.000118	0.001991
281	Lateral_Area_7A_R	0.000840	0.003587
288	Area_Lateral_IntraParietal_ventral_R	0.000097	0.001991
290	Ventral_IntraParietal_Complex_R	0.000726	0.003447
293	Area_IntraParietal_2_R	0.000968	0.003587
299	Area_PGp_R	0.000388	0.002604
301	Area_23d_R	0.000573	0.003026
309	Parieto-Occipital_Sulcus_Area_1_R	0.000932	0.003587
310	Parieto-Occipital_Sulcus_Area_2_R	0.000539	0.002952
318	Area_8BM_R	0.000058	0.001893
319	Area_9_Middle_R	0.000727	0.003447
326	Area_p32_R	0.000861	0.003587
335	Area_47s_R	0.000752	0.003489
339	Area_44_R	0.000817	0.003573
340	Area_45_R	0.000150	0.002107
341	Area_47l_(47_lateral)_R	0.000263	0.002244
344	Area_IFJp_R	0.000355	0.002511
345	Area_IFSa_R	0.000442	0.002773
349	Area_8Ad_R	0.000944	0.003587
350	Area_8Av_R	0.000691	0.003445
353	Area_9-46d_R	0.000009	0.001212
382	Putamen_L	0.000041	0.001640
415	Putamen_R	0.000817	0.003573

2.2. Rule effect in memory trial

HCPex region number	HCPex region name	p(one-tail)	FDR-p(one-tail)
Null	Null	Null	Null

2.3. Higher Rule effect in rule than memory trials

HCPex region number	HCPex region name	p(one-tail)	FDR-p(one-tail)
regionNo	Name	p	q
1	Primary_Visual_Cortex_L	0.000170	0.001336
2	Second_Visual_Area_L	0.000249	0.001686
3	Third_Visual_Area_L	0.000015	0.000456
4	Fourth_Visual_Area_L	0.000015	0.000456
5	IntraParietal_Sulcus_Area_1_L	0.000046	0.000742
9	Area_V6A_L	0.000516	0.002257
11	Fusiform_Face_Complex_L	0.000034	0.000652
13	Eighth_Visual_Area_L	0.000281	0.001711
15	VentroMedial_Visual_Area_2_L	0.000004	0.000394
17	Ventral_Visual_Complex_L	0.000481	0.002257
20	Area_Lateral_Occipital_2_L	0.000845	0.003005
24	Area_PH_L	0.000437	0.002126
26	Area_V4t_L	0.000567	0.002424
28	Area_2_L	0.000412	0.002080

30	Primary_Sensory_Cortex_L	0.000715	0.002764
31	Primary_Motor_Cortex_L	0.000021	0.000519
33	Dorsal_Area_24d_L	0.000761	0.002875
35	Area_5L_L	0.000175	0.001336
40	Supplementary_and_Cingulate_Eye_Field_L	0.000174	0.001336
42	Area_6_anterior_L	0.000417	0.002080
43	Dorsal_area_6_L	0.000014	0.000456
44	Rostral_Area_6_L	0.000355	0.001960
45	Ventral_Area_6_L	0.000011	0.000456
46	Frontal_Eye_Fields_L	0.000011	0.000456
56	Medial_Belt_Complex_L	0.000122	0.001076
57	ParaBelt_Complex_L	0.000048	0.000742
60	Auditory_4_Complex_L	0.000118	0.001066
61	Auditory_5_Complex_L	0.000033	0.000652
64	Area_STSd_posterior_L	0.000633	0.002560
66	Area_STSv_posterior_L	0.000977	0.003248
67	Area_TA2_L	0.000117	0.001066
68	Anterior_Agranular_Insula_Complex_L	0.000988	0.003258
72	Frontal_Opercular_Area_4_L	0.000402	0.002080
75	Middle_Insular_Area_L	0.000512	0.002257
79	Posterior_Insular_Area_2_L	0.000003	0.000352
80	Hippocampus_L	0.000818	0.002973
81	PreSubiculum_L	0.000187	0.001396
88	Area_PHT_L	0.000068	0.000825
89	Area_TE1_anterior_L	0.000012	0.000456
90	Area_TE1_Middle_L	0.000273	0.001686
92	Area_TE2_anterior_L	0.000052	0.000742
93	Area_TE2_posterior_L	0.000768	0.002875
94	Area_TG_dorsal_L	0.000618	0.002560
97	Superior_Temporal_Visual_Area_L	0.000064	0.000805
98	Area_TemporoParietoOccipital_Junction_1_L	0.000064	0.000805
102	Medial_Area_7A_L	0.000079	0.000879
104	Lateral_Area_7P_L	0.000506	0.002257
105	Medial_Area_7P_L	0.000833	0.003001
106	Anterior_IntraParietal_Area_L	0.000638	0.002560
107	Area_Lateral_IntraParietal_dorsal_L	0.000002	0.000352
108	Area_Lateral_IntraParietal_ventral_L	0.000070	0.000825
109	Medial_IntraParietal_Area_L	0.000097	0.000970
110	Ventral_IntraParietal_Complex_L	0.000015	0.000456
111	Area_IntraParietal_0_L	0.000038	0.000702
112	Area_IntraParietal_1_L	0.000504	0.002257
113	Area_IntraParietal_2_L	0.000273	0.001686
114	Area_PF_Complex_L	0.000535	0.002315
118	Area_PGi_L	0.000270	0.001686
119	Area_PGp_L	0.000104	0.001011
120	Area_PGs_L	0.000891	0.003067
123	Area_31pd_L	0.000270	0.001686

128	PreCuneus_Visual_Area_L	0.000014	0.000456
129	Parieto-Occipital_Sulcus_Area_1_L	0.000936	0.003156
130	Parieto-Occipital_Sulcus_Area_2_L	0.000018	0.000456
132	RetroSplenial_Complex_L	0.000091	0.000968
138	Area_8BM_L	0.000294	0.001756
142	Area_anterior_32_prime_L	0.000003	0.000352
143	Area_dorsal_32_L	0.000869	0.003041
147	Area_p32_prime_L	0.000314	0.001826
159	Area_44_L	0.000623	0.002560
160	Area_45_L	0.000487	0.002257
165	Area_IFSa_L	0.000094	0.000968
166	Area_IFSp_L	0.000391	0.002074
168	Area_46_L	0.000395	0.002074
172	Area_8C_L	0.000017	0.000456
173	Area_9-46d_L	0.000899	0.003067
175	Area_9_Posterior_L	0.000095	0.000968
177	Inferior_6-8_Transitional_Area_L	0.000192	0.001412
178	Area_posterior_9-46v_L	0.000131	0.001104
181	Primary_Visual_Cortex_R	0.000010	0.000456
183	Third_Visual_Area_R	0.000718	0.002764
184	Fourth_Visual_Area_R	0.000321	0.001836
185	IntraParietal_Sulcus_Area_1_R	0.000769	0.002875
187	Area_V3B_R	0.000706	0.002764
191	Fusiform_Face_Complex_R	0.000057	0.000768
195	VentroMedial_Visual_Area_2_R	0.000053	0.000742
197	Ventral_Visual_Complex_R	0.000633	0.002560
199	Area_Lateral_Occipital_1_R	0.000473	0.002257
205	Area_V3CD_R	0.000348	0.001960
221	Area_55b_R	0.000427	0.002101
222	Area_6_anterior_R	0.000161	0.001333
226	Frontal_Eye_Fields_R	0.000298	0.001756
227	Premotor_Eye_Field_R	0.000247	0.001686
235	Lateral_Belt_Complex_R	0.000025	0.000565
236	Medial_Belt_Complex_R	0.000223	0.001576
252	Frontal_Opercular_Area_4_R	0.000358	0.001960
255	Middle_Insular_Area_R	0.000941	0.003156
257	Pirform_Cortex_R	0.000596	0.002521
264	Area_TF_R	0.000507	0.002257
273	Area_TE2_posterior_R	0.000053	0.000742
278	Area_TemporoParietoOccipital_Junction_1_R	0.000501	0.002257
288	Area_Lateral_IntraParietal_ventral_R	0.000026	0.000565
305	Area_7m_R	0.000795	0.002917
309	Parieto-Occipital_Sulcus_Area_1_R	0.000387	0.002074
310	Parieto-Occipital_Sulcus_Area_2_R	0.000126	0.001090
312	RetroSplenial_Complex_R	0.000788	0.002917
319	Area_9_Middle_R	0.000875	0.003041
322	Area_anterior_32_prime_R	0.000850	0.003005

335	Area_47s_R	0.000268	0.001686
345	Area_IFSa_R	0.000417	0.002080
350	Area_8Av_R	0.000040	0.000715
353	Area_9-46d_R	0.000209	0.001502
357	Inferior_6-8_Transitional_Area_R	0.000253	0.001686
358	Area_posterior_9-46v_R	0.000116	0.001066
382	Putamen_L	0.000052	0.000742
383	Caudate_L	0.000676	0.002685
415	Putamen_R	0.000073	0.000832
416	Caudate_R	0.000175	0.001336

2.4. Cue effect in rule trial

HCPex region number	HCPex region name	p(one-tail)	FDR-p(one-tail)
27	Area_1_L	0.000005	0.000672
30	Primary_Sensory_Cortex_L	0.000441	0.028613
31	Primary_Motor_Cortex_L	0.000674	0.037410
43	Dorsal_area_6_L	0.000769	0.037410
207	Area_1_R	0.000005	0.000672
208	Area_2_R	0.000123	0.009582
209	Area_3a_R	0.000042	0.004105
210	Primary_Sensory_Cortex_R	0.000002	0.000672

2.5. Cue effect in memory trial

HCPex region number	HCPex region name	p(one-tail)	FDR-p(one-tail)
1	Primary_Visual_Cortex_L	0.000000	0.000012
2	Second_Visual_Area_L	0.000000	0.000005
3	Third_Visual_Area_L	0.000003	0.000048
4	Fourth_Visual_Area_L	0.000002	0.000031
11	Fusiform_Face_Complex_L	0.000074	0.000494
17	Ventral_Visual_Complex_L	0.000454	0.001900
24	Area_PH_L	0.000782	0.002714
27	Area_1_L	0.000001	0.000023
28	Area_2_L	0.000014	0.000166
29	Area_3a_L	0.000033	0.000295
30	Primary_Sensory_Cortex_L	0.000000	0.000002
31	Primary_Motor_Cortex_L	0.000000	0.000001
33	Dorsal_Area_24d_L	0.000001	0.000016
35	Area_5L_L	0.000141	0.000794
38	Area_6m_anterior_L	0.000960	0.003166
40	Supplementary_and_Cingulate_Eye_Field_L	0.000025	0.000233
41	Area_55b_L	0.000170	0.000934
42	Area_6_anterior_L	0.000136	0.000791
43	Dorsal_area_6_L	0.000017	0.000186
44	Rostral_Area_6_L	0.000906	0.003037
45	Ventral_Area_6_L	0.000060	0.000432

46	Frontal_Eye_Fields_L	0.000002	0.000037
48	Area_43_L	0.000041	0.000352
50	Area_OP1-SII_L	0.000051	0.000381
51	Area_OP2-3-VS_L	0.000633	0.002436
52	Area_OP4-PV_L	0.000613	0.002386
74	Insular_Granular_Complex_L	0.000198	0.001058
88	Area_PHT_L	0.000048	0.000381
93	Area_TE2_posterior_L	0.000447	0.001891
98	Area_TemporoParietoOccipital_Junction_1_L	0.000295	0.001382
102	Medial_Area_7A_L	0.000133	0.000784
103	Area_7PC_L	0.000017	0.000186
106	Anterior_IntraParietal_Area_L	0.000085	0.000551
107	Area_Lateral_IntraParietal_dorsal_L	0.000392	0.001694
108	Area_Lateral_IntraParietal_ventral_L	0.000002	0.000031
110	Ventral_IntraParietal_Complex_L	0.000603	0.002371
111	Area_IntraParietal_0_L	0.000559	0.002265
112	Area_IntraParietal_1_L	0.000654	0.002447
113	Area_IntraParietal_2_L	0.000286	0.001355
114	Area_PF_Complex_L	0.000000	0.000000
115	Area_PFm_Complex_L	0.000001	0.000023
116	Area_PF_Opercular_L	0.000332	0.001501
117	Area_PFt_L	0.000225	0.001166
119	Area_PGp_L	0.000005	0.000070
120	Area_PGs_L	0.000279	0.001355
121	Area_23d_L	0.000678	0.002512
122	Area_31a_L	0.000175	0.000945
123	Area_31pd_L	0.000060	0.000432
124	Area_31p_ventral_L	0.000130	0.000778
125	Area_7m_L	0.000020	0.000200
126	Area_dorsal_23_a+b_L	0.000697	0.002535
128	PreCuneus_Visual_Area_L	0.000046	0.000381
130	Parieto-Occipital_Sulcus_Area_2_L	0.000003	0.000052
142	Area_anterior_32_prime_L	0.000652	0.002447
159	Area_44_L	0.000789	0.002715
160	Area_45_L	0.000020	0.000200
161	Area_47l_(47_lateral)_L	0.000874	0.002982
165	Area_IFSa_L	0.000116	0.000706
172	Area_8C_L	0.000215	0.001132
177	Inferior_6-8_Transitional_Area_L	0.000088	0.000552
178	Area_posterior_9-46v_L	0.000504	0.002063
181	Primary_Visual_Cortex_R	0.000000	0.000000
182	Second_Visual_Area_R	0.000000	0.000002
183	Third_Visual_Area_R	0.000002	0.000037
184	Fourth_Visual_Area_R	0.000002	0.000032
185	IntraParietal_Sulcus_Area_1_R	0.000367	0.001621
188	Sixth_Visual_Area_R	0.000005	0.000064
190	Seventh_Visual_Area_R	0.000426	0.001823

192	Posterior_InferoTemporal_complex_R	0.000731	0.002588
193	Eighth_Visual_Area_R	0.000049	0.000381
196	VentroMedial_Visual_Area_3_R	0.000007	0.000090
199	Area_Lateral_Occipital_1_R	0.000078	0.000517
200	Area_Lateral_Occipital_2_R	0.000599	0.002371
203	Middle_Temporal_Area_R	0.000994	0.003230
205	Area_V3CD_R	0.000235	0.001203
206	Area_V4t_R	0.000285	0.001355
207	Area_1_R	0.000000	0.000013
208	Area_2_R	0.000004	0.000062
209	Area_3a_R	0.000000	0.000004
210	Primary_Sensory_Cortex_R	0.000000	0.000003
211	Primary_Motor_Cortex_R	0.000001	0.000015
213	Dorsal_Area_24d_R	0.000000	0.000013
215	Area_5L_R	0.000247	0.001248
216	Area_5m_R	0.000707	0.002548
218	Area_6m_anterior_R	0.000376	0.001645
219	Area_6mp_R	0.000068	0.000472
223	Dorsal_area_6_R	0.000018	0.000186
225	Ventral_Area_6_R	0.000107	0.000658
226	Frontal_Eye_Fields_R	0.000001	0.000015
230	Area_OP1-SII_R	0.000000	0.000000
258	Area_Posterior_Insular_1_R	0.000732	0.002588
266	ParaHippocampal_Area_2_R	0.000256	0.001275
278	Area_TemporoParietoOccipital_Junction_1_R	0.000015	0.000182
280	Area_TemporoParietoOccipital_Junction_3_R	0.000156	0.000868
281	Lateral_Area_7A_R	0.000648	0.002447
282	Medial_Area_7A_R	0.000341	0.001523
283	Area_7PC_R	0.000044	0.000372
284	Lateral_Area_7P_R	0.000026	0.000237
286	Anterior_IntraParietal_Area_R	0.000687	0.002522
287	Area_Lateral_IntraParietal_dorsal_R	0.000467	0.001933
288	Area_Lateral_IntraParietal_ventral_R	0.000061	0.000432
289	Medial_IntraParietal_Area_R	0.000000	0.000000
291	Area_IntraParietal_0_R	0.000276	0.001355
292	Area_IntraParietal_1_R	0.000317	0.001449
293	Area_IntraParietal_2_R	0.000004	0.000062
294	Area_PF_Complex_R	0.000051	0.000381
295	Area_PFm_Complex_R	0.000029	0.000266
300	Area_PGs_R	0.000017	0.000186
302	Area_31a_R	0.000739	0.002589
304	Area_31p_ventral_R	0.000310	0.001435
305	Area_7m_R	0.000595	0.002371
306	Area_dorsal_23_a+b_R	0.000012	0.000148
310	Parieto-Occipital_Sulcus_Area_2_R	0.000893	0.003022
348	Area_46_R	0.000921	0.003061
350	Area_8Av_R	0.000021	0.000200

357	Inferior_6-8_Transitional_Area_R	0.000086	0.000551
358	Area_posterior_9-46v_R	0.000050	0.000381
381	Thal_Ventral_posterolateral_L	0.000139	0.000794
382	Putamen_L	0.000074	0.000494
415	Putamen_R	0.000998	0.003230

2.6. Higher Cue effect in memory than rule trials

HCPex region number	HCPex region name	p(one-tail)	FDR-p(one-tail)
2	Second_Visual_Area_L	0.000405	0.003941
3	Third_Visual_Area_L	0.000080	0.001475
4	Fourth_Visual_Area_L	0.000001	0.000110
5	IntraParietal_Sulcus_Area_1_L	0.000903	0.006388
11	Fusiform_Face_Complex_L	0.000084	0.001477
15	VentroMedial_Visual_Area_2_L	0.000019	0.000695
26	Area_V4t_L	0.000764	0.005943
35	Area_5L_L	0.000201	0.002796
44	Rostral_Area_6_L	0.000661	0.005382
49	Frontal_Opercular_Area_1_L	0.000274	0.003553
64	Area_STSd_posterior_L	0.000033	0.000906
81	PreSubiculum_L	0.000080	0.001475
85	ParaHippocampal_Area_1_L	0.000181	0.002604
88	Area_PHT_L	0.000119	0.002013
93	Area_TE2_posterior_L	0.000664	0.005382
98	Area_TemporoParietoOccipital_Junction_1_L	0.000173	0.002581
103	Area_7PC_L	0.000222	0.002974
107	Area_Lateral_IntraParietal_dorsal_L	0.000702	0.005573
108	Area_Lateral_IntraParietal_ventral_L	0.000000	0.000110
110	Ventral_IntraParietal_Complex_L	0.000003	0.000302
111	Area_IntraParietal_0_L	0.000445	0.004123
119	Area_PGp_L	0.000326	0.003941
122	Area_31a_L	0.000171	0.002581
125	Area_7m_L	0.000658	0.005382
128	PreCuneus_Visual_Area_L	0.000006	0.000466
130	Parieto-Occipital_Sulcus_Area_2_L	0.000885	0.006388
142	Area_anterior_32_prime_L	0.000436	0.004123
160	Area_45_L	0.000015	0.000695
165	Area_IFSa_L	0.000031	0.000906
166	Area_IFSp_L	0.000003	0.000302
172	Area_8C_L	0.000901	0.006388
178	Area_posterior_9-46v_L	0.000377	0.003941
181	Primary_Visual_Cortex_R	0.000015	0.000695
182	Second_Visual_Area_R	0.000021	0.000695
184	Fourth_Visual_Area_R	0.000050	0.001206
185	IntraParietal_Sulcus_Area_1_R	0.000315	0.003941
191	Fusiform_Face_Complex_R	0.000393	0.003941
193	Eighth_Visual_Area_R	0.000378	0.003941

195	VentroMedial_Visual_Area_2_R	0.000021	0.000695
196	VentroMedial_Visual_Area_3_R	0.000522	0.004509
197	Ventral_Visual_Complex_R	0.000017	0.000695
199	Area_Lateral_Occipital_1_R	0.000055	0.001267
200	Area_Lateral_Occipital_2_R	0.000501	0.004434
202	Medial_Superior_Temporal_Area_R	0.000992	0.006727
204	Area_PH_R	0.000353	0.003941
227	Premotor_Eye_Field_R	0.000362	0.003941
282	Medial_Area_7A_R	0.000369	0.003941
284	Lateral_Area_7P_R	0.000156	0.002529
288	Area_Lateral_IntraParietal_ventral_R	0.000043	0.001126
289	Medial_IntraParietal_Area_R	0.000020	0.000695
290	Ventral_IntraParietal_Complex_R	0.000463	0.004188
291	Area_IntraParietal_0_R	0.000073	0.001475
300	Area_PGs_R	0.000841	0.006388
312	RetroSplenial_Complex_R	0.000900	0.006388
379	Thal_Ventral_Lateral_Anterior_L	0.000400	0.003941
382	Putamen_L	0.000078	0.001475

2.7. Double dissociation effect (Conjunction)

HCPex region number	HCPex region name	p(one-tail)	FDR-p(one-tail)
2	Second_Visual_Area_L	0.000010	0.000085
3	Third_Visual_Area_L	0.000000	0.000011
4	Fourth_Visual_Area_L	0.000000	0.000008
5	IntraParietal_Sulcus_Area_1_L	0.000003	0.000040
11	Fusiform_Face_Complex_L	0.000001	0.000019
15	VentroMedial_Visual_Area_2_L	0.000000	0.000006
26	Area_V4t_L	0.000037	0.000194
35	Area_5L_L	0.000006	0.000059
44	Rostral_Area_6_L	0.000018	0.000125
64	Area_STSd_posterior_L	0.000004	0.000044
81	PreSubiculum_L	0.000003	0.000043
88	Area_PHT_L	0.000002	0.000026
93	Area_TE2_posterior_L	0.000045	0.000223
98	Area_TemporoParietoOccipital_Junction_1_L	0.000001	0.000018
107	Area_Lateral_IntraParietal_dorsal_L	0.000000	0.000008
108	Area_Lateral_IntraParietal_ventral_L	0.000001	0.000016
110	Ventral_IntraParietal_Complex_L	0.000000	0.000008
111	Area_IntraParietal_0_L	0.000001	0.000023
119	Area_PGp_L	0.000004	0.000044
128	PreCuneus_Visual_Area_L	0.000000	0.000008
130	Parieto-Occipital_Sulcus_Area_2_L	0.000001	0.000016
142	Area_anterior_32_prime_L	0.000000	0.000007
160	Area_45_L	0.000009	0.000081
165	Area_IFSa_L	0.000001	0.000016
166	Area_IFSp_L	0.000003	0.000041

172	Area_8C_L	0.000001	0.000016
178	Area_posterior_9-46v_L	0.000004	0.000048
181	Primary_Visual_Cortex_R	0.000000	0.000006
184	Fourth_Visual_Area_R	0.000010	0.000085
185	IntraParietal_Sulcus_Area_1_R	0.000030	0.000172
191	Fusiform_Face_Complex_R	0.000002	0.000036
195	VentroMedial_Visual_Area_2_R	0.000001	0.000016
197	Ventral_Visual_Complex_R	0.000014	0.000108
199	Area_Lateral_Occipital_1_R	0.000017	0.000122
227	Premotor_Eye_Field_R	0.000014	0.000109
288	Area_Lateral_IntraParietal_ventral_R	0.000000	0.000013
312	RetroSplenial_Complex_R	0.000042	0.000213
382	Putamen_L	0.000001	0.000018
