# Supplementary material for: Cost-benefit tradeoff mediates the transition from rule-based to memory-based processing during practice
Source: PLoS Biol. 2025 Jan 23;23(1):e3002987. doi: 10.1371/journal.pbio.3002987 (PMC11793810; doi:10.1371/journal.pbio.3002987)
Supplement: S1 Text — Note A. Behavioral Analysis with All Cued Positions. Note B. No Evidence Supporting the Discount of Rule-based Strategy Cost in the Cost-benefit Analysis. Note C. No Evidence Supporting Backward Replay. Note D. Detailed Task Design in the Training Phase. Note E. fMRI Data Preprocessing. Note F. Univariate Evidence for Rule- and Memory-based Strategies. Note G. Detailed RSA Methods. Note H. Evidence of Replay in Rule-based Task Retrieval. (DOCX) [file pbio.3002987.s002.docx]

## Notes

### **Note A.** Behavioral Analysis with All Cued Positions.

We observed that cued position 5 showed better performance compared to cued position 4 and that cued position 1 outperformed all other cued positions. These are possibly due to better memory of the first and last items of a sequence ^1^. To test the robustness of the behavioral findings, we repeated the mixed-effect model analyses in the main text while including all five cued positions. That is, we used cued position (1–5), block (1–6) and their interaction as predictors of performance. We found similar results as reported in the main text. Specifically, the RT results showed a significant main effect of block (*β* = –0.36, SE = 0.03, 95% confidence interval (CI) = [–0.42 –0.31], *t*(33.0) = –13.80, *p* < .001, η*_p_*^2^ = .27), a significant main effect of position (*β* = 0.29, SE = 0.04, 95% CI = [0.21 0.37], *t*(33.0) = 7.41, *p* < .001, η*_p_*^2^ = .19), and a significant interaction effect (*β* = –0.09, SE = 0.02, 95% CI = [–0.13 –0.05], *t*(580.8) = –4.47, *p* < .001, η*_p_*^2^ = .02). Post-hoc comparisons showed that the slope of position in block 1 was larger than block 3, 4, 5, and 6, whereas block 2 was larger than block 6, *p*s < .05.­ The ER results showed a significant main effect of block (*β* = –0.23, SE = 0.05, 95% CI = [–0.33 –0.13], *t*(33.0) = –4.78, *p* < .001, η*_p_*^2^ = .07), position (*β* = 0.16, SE = 0.04, 95% CI = [0.08 0.24], *t*(38.8) = 4.09, *p* < .001, η*_p_*^2^ = .04), and a significant interaction effect (*β* = –0.13, SE = 0.04, 95% CI = [–0.21 –0.05], *t*(39.6) = –3.45, *p* < .001, η*_p_*^2^ = .02). Post-hoc comparisons suggested that the slope of position in block 1 was larger than block 3, 4, and 6, whereas block 2 was larger than block 3 and 6, and block 4 and block 5 were larger than block 6, *p*s < .05. These results suggest that our behavioral findings were not qualitatively affected by the exclusion of cued position 5.

### **Note B.** No Evidence Supporting the Discount of Rule-based Strategy Cost in the Cost-benefit Analysis

With a new model (M6), we tested the possibility that practicing the rule-based strategy would reduce its cost in the cost-benefit analysis. To this end, the rule-based strategy cost was multiplied by a discount factor (D), which takes the following form:

D = $\text{e}^{\delta\text{N}}$,

where N denotes the number of experienced rule trials (including those occurring during the transition test in the training phase). *δ* is a free parameter that ranges from –1000 to 0, which simulates a wide range of decrease trajectories, including non-linear decrease and quasi-linear decrease with various slopes.

M6 was constructed by adding the discount factor to our previously validated model (M0). Model fitting resulted in a group sum Akaike information criterion (AIC) of –3928, higher than that of M0 (–4019), *p* < .001. This result suggests that the acceleration of sequence recall with practice does not significantly affect the cost-benefit analysis.

### **Note C.** No Evidence Supporting Backward Replay

We investigated whether participants engaged in backward replay process ^2^ (i.e., starting by recalling the 5^th^ position and then transitioning backward until the cued position). One might intuitively expect this to occur for later positions such as A4, since executing a backward replay only requires one step, whereas a forward replay demands three steps. However, given that our training exclusively focused on forward transitions, it remains unclear whether participants would spontaneously adopt backward replay strategies during testing. To address this question, we developed a new model (M7). With this model, we included in the cost-benefit analysis the value of implementing backward replay for each trial. To capture the individual differences in the cost of backward replay, the cost per replaying step of backward replay is that of the forward replay scaled by a factor of $\frac{1}{\mu}$, where $\mu$ ranges from 0 to 1. In other words, for a single step of replay, the cost of backward replay ranges from the cost of forward replay (when *μ* = 1) to infinite (when *μ* = 0). We opted for this implementation under the assumption that backward replay is more costly than or as costly as forward replay. Our results showed that this model performed worse (AIC = –3984) compared to M0 (AIC = –4019), *p* < .001. Further examination of M7 revealed that, on average, participants were estimated to engage in backward replay for 5.9 trials, whereas forward replay was implemented for 80.8 trials, suggesting that, even if backward replay is employed, it is far less prevalent among participants than forward replay.

### **Note D.** Detailed Task Design in the Training Phase.

The training task was programmed with JsPsych (version 7.0, https://www.jspsych.org/) and conducted online. The training task aimed to facilitate participants in learning the transitions within two task sequences. At the beginning of each mini-block, participants were instructed to equip an avatar based on a goal image (Fig 1A) in five steps (trials). We used two sequences, coded A and B, with the order of a→b→c→d→e and d→c→a→e→b, respectively. Each lowercase letter represents an equipment type (HELMET, VEST, GLASSES, WEAPON, or TOOL). The letter-equipment type mapping was randomly chosen for each participant. For each mini-block, the goal image was a fully equipped avatar with all gears randomly selected, so that the participants would learn the order of equipment type rather than specific gears.

Each sequence started with the presentation of a sequence cue (A or B) for 2s. Within the sequence, each trial started with a fixation cross for 2s and followed by the presentation of the goal image for 1s. After a blank screen of 0.5s, a partially equipped avatar (representing current progress) and two gears of the same type were presented. Participants were required to choose the gear from the goal image using a button press (left or right) within 2.5s, followed by feedback. The same trial was repeated if an error was committed. After the fifth trial, a “goal achieved” screen was shown for 2s to signal the end of the mini-block.

Following each mini-block, we tested the participant’s memory of task transitions with two test trials (S1 Fig). On each test trial, participants needed to choose the 1^st^/2^nd^/3^rd^ task following a cued task. The cued task was selected randomly from the first four tasks from each sequence. The number of transitions (1, 2, or 3) was randomly sampled from a geometric distribution (p = 0.5). Participants responded using keys “Q”, “W”, “O” and “P” with no time limit. Incorrect responses were followed by feedback and repetition of the test trial. The average accuracy for the test trials was displayed at the end of each block.

Participants performed 3-6 blocks of 12 mini-blocks each until their block-average accuracy of test trials reached 90% in the last block. Participants who could not meet the 90% accuracy after six blocks were not scanned. Thirty-five participants underwent the training phase 4-24 hours before scanning. They repeated the training phase before scanning until they could reach the 90% accuracy threshold again (all completed within 2 blocks). One participant completed the training phase immediately before scanning.

### **Note E.** fMRI Data Preprocessing

Results included in this manuscript come from preprocessing performed using fMRIPrep 22.0.2 (RRID:SCR_016216) ^3,4^, which is based on Nipype 1.8.5 (RRID:SCR_002502) ^5,6^.

*Preprocessing of B0 inhomogeneity mappings.* A B0-nonuniformity map (or fieldmap) was estimated based on two (or more) echo-planar imaging (EPI) references with topup (FSL 6.0.5.1:57b01774) ^7^.

*Anatomical data preprocessing.* The T1-weighted (T1w) image was corrected for intensity non-uniformity (INU) with N4BiasFieldCorrection ^8^, distributed with ANTs 2.3.3 (RRID:SCR_004757) ^9^, and used as T1w-reference throughout the workflow. The T1w-reference was then skull-stripped with a Nipype implementation of the antsBrainExtraction.sh workflow (from ANTs), using OASIS30ANTs as target template. Brain tissue segmentation of cerebrospinal fluid (CSF), white-matter (WM) and gray-matter (GM) was performed on the brain-extracted T1w using fast (FSL 6.0.5.1:57b01774, RRID:SCR_002823) ^10^. Volume-based spatial normalization to one standard space (MNI152NLin2009cAsym) was performed through nonlinear registration with antsRegistration (ANTs 2.3.3), using brain-extracted versions of both T1w reference and the T1w template. The following template was selected for spatial normalization: ICBM 152 Nonlinear Asymmetrical template version 2009c [RRID:SCR_008796; TemplateFlow ID: MNI152NLin2009cAsym] ^11^.

*Functional data preprocessing.* For each of the 6 BOLD runs, the following preprocessing was performed. First, a reference volume and its skull-stripped version were generated using a custom methodology of fMRIPrep. Head-motion parameters with respect to the BOLD reference (transformation matrices, and six corresponding rotation and translation parameters) are estimated before any spatiotemporal filtering using mcflirt (FSL 6.0.5.1:57b01774) ^12^. The estimated fieldmap was then aligned with rigid-registration to the target EPI (echo-planar imaging) reference run. The field coefficients were mapped on to the reference EPI using the transform. The BOLD reference was then co-registered to the T1w reference using mri_coreg (FreeSurfer) followed by flirt (FSL 6.0.5.1:57b01774) ^13^ with the boundary-based registration ^14^ cost-function. Co-registration was configured with six degrees of freedom. Several confounding time-series were calculated based on the preprocessed BOLD: framewise displacement (FD), DVARS and three region-wise global signals. FD was computed using two formulations following Power (absolute sum of relative motions) ^15^ and Jenkinson (relative root mean square displacement between affines) ^12^. FD and DVARS are calculated for each functional run, both using their implementations in Nipype (following the definitions by ^15^). The three global signals are extracted within the CSF, the WM, and the whole-brain masks. Additionally, a set of physiological regressors were extracted to allow for component-based noise correction (CompCor) ^16^. Principal components are estimated after high-pass filtering the preprocessed BOLD time-series (using a discrete cosine filter with 128s cut-off) for the two CompCor variants: temporal (tCompCor) and anatomical (aCompCor). tCompCor components are then calculated from the top 2% variable voxels within the brain mask. For aCompCor, three probabilistic masks (CSF, WM and combined CSF+WM) are generated in anatomical space. The implementation differs from that of Behzadi et al. in that instead of eroding the masks by 2 pixels on BOLD space, a mask of pixels that likely contain a volume fraction of GM is subtracted from the aCompCor masks. This mask is obtained by thresholding the corresponding partial volume map at 0.05, and it ensures components are not extracted from voxels containing a minimal fraction of GM. Finally, these masks are resampled into BOLD space and binarized by thresholding at 0.99 (as in the original implementation). Components are also calculated separately within the WM and CSF masks. For each CompCor decomposition, the k components with the largest singular values are retained, such that the retained components’ time series are sufficient to explain 50 percent of variance across the nuisance mask (CSF, WM, combined, or temporal). The remaining components are dropped from consideration. The head-motion estimates calculated in the correction step were also placed within the corresponding confounds file. The confound time series derived from head motion estimates and global signals were expanded with the inclusion of temporal derivatives and quadratic terms for each ^17^. Frames that exceeded a threshold of 0.5 mm FD or 1.5 standardized DVARS were annotated as motion outliers. Additional nuisance timeseries are calculated by means of principal components analysis of the signal found within a thin band (crown) of voxels around the edge of the brain, as proposed by ref ^18^. The BOLD time-series were resampled into standard space, generating a preprocessed BOLD run in MNI152NLin2009cAsym space. First, a reference volume and its skull-stripped version were generated using a custom methodology of fMRIPrep. All resamplings can be performed with a single interpolation step by composing all the pertinent transformations (i.e., head-motion transform matrices, susceptibility distortion correction when available, and co-registrations to anatomical and output spaces). Gridded (volumetric) resamplings were performed using antsApplyTransforms (ANTs), configured with Lanczos interpolation to minimize the smoothing effects of other kernels ^19^. Non-gridded (surface) resamplings were performed using mri_vol2surf (FreeSurfer).

Many internal operations of fMRIPrep use Nilearn 0.9.1 (RRID:SCR_001362) ^20^, mostly within the functional processing workflow. For more details of the pipeline, see the section corresponding to workflows in fMRIPrep’s documentation.

*Copyright Waiver.* The above boilerplate text was automatically generated by fMRIPrep with the express intention that users should copy and paste this text into their manuscripts unchanged. It is released under the CC0 license.

### **Note F.** Univariate Evidence for Rule- and Memory-based Strategies.

To detect the brain regions whose fMRI activation reflects key components of the two strategies, we conducted three LME models with ROI-averaged fMRI activation levels. The first LME model was conducted on rule trials and incorporated several fixed effect regressors, including the rule implementation cost C, C*Times_R_ (to account for the acceleration of rule-based strategy due to practice), and the logarithm of trial number (to account for strategy-agnostic practice effect). The second LME model was conducted on memory trials and included fixed effect regressors of memory strength and the logarithm of trial number. The third LME model was conducted on both rule and memory trials and included fixed effect regressors of the decision variable (defined by the utility difference of alternative strategies, log-transformed), C, C*Times_R_, memory strength, and the logarithm of trial number. The first two models also included an intercept, and the third model included two intercepts indicating the two trial types. Additionally, the intercepts and the slope of each regressor were also treated as random effects at the subject level. All predictors were standardized. Statistical results were corrected for multiple comparisons across all the ROIs with false discovery rate (FDR) at the level of *q* < 0.05, with each individual ROI at uncorrected *p* < .001, both one-tailed for the rule trials and two-tailed for the memory trials based on our different hypotheses (see below).

We predicted stronger fMRI activation when later positions were cued on rule trials, possibly due to increased effort in implementing the rule-based strategy (e.g., the steps of replay ^21^, difficulty, and working memory load). Higher rule implementation costs were accompanied by stronger activation in several frontal regions, including Human Connectome Project (HCP) Atlas ^22^ areas 8C, anterior inferior frontal junction (IFJa), posterior 47 rostral (p47r), anterior 47 rostral (a47r), anterior 9-46 ventral (a9-46v), posterior 32 prime (p32pr), inferior 6-8 (i6-8) in the left hemisphere and 8C, posterior 9-46 ventral (p9-46v), 44 in the right hemisphere, *p*s < .05 corrected (Figs S3A-B). On memory trials, fMRI activation may be stronger when the cue-task association is stronger (reflecting the memory retrieval effect) ^23^ or weaker (reflecting the uncertainty effect) ^24^. We found stronger brain activation with stronger memory of the cued position-task association in the left PF (Figs S3C-D, *p* < .05 corrected), and stronger brain activation with weaker memory of the cued position-task association in the left supplementary and cingulate eye field (SCEF) and the left posterior IFJ (IFJp) (Figs S3E-F). No other regions survived the correction for multiple comparisons. However, the decision variable was not associated with any specific brain regions, suggesting that the encoding of the cost-benefit tradeoff in the dorsomedial prefrontal cortex might only occur in a distributed fashion (Fig 5C). These results are consistent with previous findings ^24-26^ and provide a sanity check of the fMRI data.

### **Note G.** Detailed RSA Methods.

*Analysis 1: Strategy-specific Representations on Rule and Memory Trials.*

The computational model categorized trials into rule and memory trials based on behavioral data. We then tested if this categorization accounted for the fMRI data. If the cost-benefit analysis decides which strategy to implement, we would expect a stronger rule effect on the rule than the memory trials and a stronger memory effect on memory than rule trials. To this end, we conducted RSA including the two factors (rule/memory effects as illustrated in Fig 3 and rule/memory trials classified by the computational model). The RSA measures the fMRI activation pattern similarity between two trials. This measure serves as the dependent variable. The key independent variables include the rule and memory effects encoding hypothetical similarity based on the corresponding strategy. Specifically, the rule effect is operationalized as the number of shared tasks between two trials (counting from the beginning of the sequence) excluding the cued task (Fig 3B). The memory effect encodes whether the two trials share the same cue, which captures the memory strategy (i.e., directly retrieving the answer from the cue without engaging the rule-based strategy, Fig 3C). Each effect was coded separately within each trial type to test the difference in the effect between rule and memory trials. To account for the potential context effect (i.e., a higher similarity for within-sequence trial pairs than across-sequence trial pairs), trial pairs were further divided into within-sequence and across-sequence trial pairs. We further added separate intercepts to each trial type. As a result, the model is formulated as follows:

RSM_brain_ = β_0_R1 + β_1_R2+ β_2_M1+ β_3_M2+ β_4_RSM_Rule_R_ + β_5_RSM_Rule_M_ + β_6_RSM_Cue_R1_ + β_7_RSM_Cue_R2_ + β_8_RSM_Cue_M1_ + β_9_RSM _Cue_M2_ + β_10_RSM_Univoxel_trial1_ + β_11_RSM_Univoxel_trial2_ + (R1|subject) + (R2|subject) + (M1|subject) + (M2|subject) + RandomSlopes + ε.

In this equation, RSM_brain_ represents the representational similarity matrix (RSM) derived from fMRI activation patterns. M and R encode memory and rule trials, respectively. Rule and Cue in subscript encode their corresponding effects. The division of trial pairs based on the context is reflected by the R1 (rule trial pairs from the same sequence), R2 (rule trial pairs from different sequences), M1 (memory trial pairs from the same sequence), and M2 (memory trial pairs from different sequences), respectively. These regressors were added to account for potential differences in the pattern similarity between same- and different-sequence trial pairs and between different trial types. RSM_Rule_ and RSM_Cue_ encode hypothesized pattern similarity levels based on rule- and memory-based strategies, respectively (Figs 3B-C). The term RandomSlopes represents the collection of random effects for the slope of each fixed effect. Additionally, the RSM_Univoxel_trial1_ and RSM_Univoxel_trial2_ capture the univoxel activation for each trial of the pair. All random effects were set at the subject level. An illustration of this model can be found in S5 Fig.

To further test the difference in rule and memory effects between the rule trials and memory trials from the same sequence, we conducted three contrast analyses. These included (1) testing if the rule effect was stronger in rule trials compared to memory trials (β_4_ > β_5_), (2) testing if the cue effect from the same sequence was stronger in memory trials compared to rule trials (β_8_ > β_6_), and (3) testing if there were regions showing a double dissociation between rule/memory strategy effect and trial type using a conjunction analysis of (1) and (2).

*Analysis 2: fMRI Boundary Effects around Transition Points*

This analysis aimed to assess changes in activation patterns during strategy transition. As in the behavioral and univariate analyses, here we focused on the two trials immediately adjacent to a transition point. We denoted the transition point at t and coded the last rule trial as t–1 and the first memory trial as t+1. We computed the similarity between these two trials and each of the trials 2–6 steps before/after the transition point (baseline trials). We hypothesized a decrease in pattern similarity when trials were from opposite sides of the boundary (i.e., transition point), such that trial t–1 would show greater similarity to the rule baseline trials (within trial type) compared to the memory baseline trials (between trial types), and vice versa for trial t+1. Thus, we constructed a regressor representing whether two trials are of the same trial type (RSM_Trialtype_). To account for the potential confound of temporal proximity, we included a nuisance regressor encoding temporal distance between each trial pair (RSM_Distance_). As above, trial-wise univariate activation levels were included as additional nuisance regressors. The resulting LME model takes the following form:

RSM_brain_ = β_0_+ β_1_RSM_Trialtype_ + β_2_RSM_Distance_ + β_3_RSM_Univoxel_trial1_ + β_4_RSM_Univoxel_trial2_ + RandomSlopes + ε.

All trial pairs in this analysis were limited to different runs to control for RSA confounds from within-run trial pairs while limiting the analysis to trials close to transition points ^27^.

*Analysis 3: Pattern Separation.*

The use of the memory strategy predicted that the learning of cue-task associations would prompt distinctive patterns to emerge, effectively separating the conditions ^28^. To test this prediction of increased pattern separation with practice, we computed the representational similarity between trial pairs that were the same temporal distance away from their respective transition points. To better capture the temporal trajectory of pattern separation for each cue, the temporal distance between a trial and its transition point counts only trials with the same cue. To test potential changes in the degree of pattern separation before and after strategy transition, we introduced two separate regressors in our model: one for rule trials and another for memory trials. As above, we also added the nuisance regressors of trial distance and trial-wise univariate activations to the LME model:

RSM_brain_ = β_0_R + β_1_M + β_2_RSM_R_ + β_3_RSM_M_ + β_4_RSM_Distance_ + β_5_RSM_Univoxel_trial1_ + β_6_RSM_Univoxel_trial2_ + RandomSlopes + ε.

This analysis focused on the average effect of trial number across both rule (β_2_) and memory (β_3_) trials. This allowed us to assess the level of pattern separation in relation to the temporal distance to the transition points. We also tested β_2_- β_3_ to explore whether the speed of pattern separation changed following strategy transition.

### **Note H.** Evidence of Replay in Rule-based Task Retrieval.

We hypothesized that rule-based processing involves replaying tasks within the same sequence to facilitate the retrieval of the cued task. To test this, we examined whether tasks preceding the cued position could be decoded above chance level through a decoding analysis. For each task, we trained a lasso logistic regression classifier to predict whether the task was replayed on each trial (i.e., from the beginning of the sequence to the cued task), setting the regularization coefficient at 0.006 ^29^. To mitigate overfitting, the leave-one-block-out cross-validation was applied. The trained classifiers were then tested on the left-out data to obtain a probabilistic prediction of the presence of each task on each trial. As a result, given the fMRI activation pattern of a trial, the classifier produced a probabilistic prediction for each task being replayed. We used these predictions to perform two further regression analyses:

The first analysis aimed to examine if the replayed tasks could be decoded (i.e., showing a higher predicted probability of being replayed than chance level) from rule trials but not memory trials. We conducted two separate analyses for the two trial types using the same formula but different dependent variables:

Probability_prediction (R or M) = β_0_ + β_1_Replayed_tasks + β_2_Cued_task + β_3_Other_task + RandomSlopes + ε,

where Probability_prediction is a trial × task matrix containing probability values, Replayed_tasks is a binary predictor indicating the hypothesized replayed tasks (i.e., tasks prior to the cued position) on each trial, Cued_task denotes the cued task, and Other_task is another binary predictor denoting the tasks posterior to the cued position.

To avoid potential bias in the decoding process, we established a baseline decoding performance by conducting permutation tests. Permutations were done by randomly shuffling the labels (e.g., A4) of each trial 500 times. Results showed that, for the 63 regions showing rule implementation representations (Fig 4A), 4 regions (left STSd_posterior, left TE1_anterior, left IFSa, and right TE2_posterior) showed a significant Replayed_tasks effect on rule trials (*p*s < .05, FDR corrected), suggesting that the hypothesized replayed tasks can be decoded above chance level. In comparison, no regions showed a Replayed_tasks effect on memory trials, *p*s = 1, uncorrected.

To further test whether the Replayed_tasks effect was stronger on rule trials than on memory trials, we conducted an interaction analysis with the following formula:

Probability_prediction = β_0_ + β_1_Replayed_tasks * Trialtype + β_2_Cued_task * Trialtype + RandomSlopes + ε,

where Trialtype is a categorical regressor denoting the trial type (1 for rule-based trials and –1 for memory-based trials). Results showed that, among the 108 regions identified in Fig 4C, eighty-one regions showed this effect, *p*s < .05, FDR corrected (S7A Fig). Across the whole-brain regions, we also identified similar brain distributions with FDR correction (S7B Fig).

## References

1. Murdock, B.B. (1962). The serial position effect of free recall. Journal of Experimental Psychology *64*, 482-488. 10.1037/h0045106.

2. Foster, D.J., and Wilson, M.A. (2006). Reverse replay of behavioural sequences in hippocampal place cells during the awake state. Nature *440*, 680-683. 10.1038/nature04587.

3. Esteban, O., Markiewicz, C.J., Blair, R.W., Moodie, C.A., Isik, A.I., Erramuzpe, A., Kent, J.D., Goncalves, M., DuPre, E., Snyder, M., et al. (2019). fMRIPrep: a robust preprocessing pipeline for functional MRI. Nat Methods *16*, 111-+. 10.1038/s41592-018-0235-4.

4. Esteban, Oscar, Blair, R., Markiewicz, C.J., Berleant, S.L., Moodie, C., Ma, F., and Ayse Ilkay Isik, e.a. (2018). FMRIPrep (Software).

5. Gorgolewski, K., Burns, C.D., Madison, C., Clark, D., Halchenko, Y.O., Waskom, M.L., and Ghosh, S.S. (2011). Nipype: a flexible, lightweight and extensible neuroimaging data processing framework in python. Frontiers in neuroinformatics *5*, 13. 10.3389/fninf.2011.00013.

6. Gorgolewski, J., K., Esteban, O., Markiewicz, C.J., Ziegler, E., Ellis, D.G., Notter, M.P., and al., D.J.e. (2018). Nipype (Software).

7. Andersson, J.L., Skare, S., and Ashburner, J. (2003). How to correct susceptibility distortions in spin-echo echo-planar images: application to diffusion tensor imaging. Neuroimage *20*, 870-888. 10.1016/S1053-8119(03)00336-7.

8. Tustison, N.J., Avants, B.B., Cook, P.A., Zheng, Y., Egan, A., Yushkevich, P.A., and Gee, J.C. (2010). N4ITK: improved N3 bias correction. IEEE Trans Med Imaging *29*, 1310-1320. 10.1109/TMI.2010.2046908.

9. Avants, B.B., Epstein, C.L., Grossman, M., and Gee, J.C. (2008). Symmetric diffeomorphic image registration with cross-correlation: evaluating automated labeling of elderly and neurodegenerative brain. Med Image Anal *12*, 26-41. 10.1016/j.media.2007.06.004.

10. Zhang, Y., Brady, M., and Smith, S. (2001). Segmentation of brain MR images through a hidden Markov random field model and the expectation-maximization algorithm. IEEE Trans Med Imaging *20*, 45-57. 10.1109/42.906424.

11. Fonov, V.S., Evans, A.C., McKinstry, R.C., Almli, C.R., and Collins, D.L. (2009). Unbiased nonlinear average age-appropriate brain templates from birth to adulthood. NeuroImage *47*. 10.1016/s1053-8119(09)70884-5.

12. Jenkinson, M., Bannister, P., Brady, M., and Smith, S. (2002). Improved optimization for the robust and accurate linear registration and motion correction of brain images. Neuroimage *17*, 825-841. 10.1016/s1053-8119(02)91132-8.

13. Jenkinson, M., and Smith, S. (2001). A global optimisation method for robust affine registration of brain images. Medical Image Analysis *5*, 143-156. 10.1016/s1361-8415(01)00036-6.

14. Greve, D.N., and Fischl, B. (2009). Accurate and robust brain image alignment using boundary-based registration. Neuroimage *48*, 63-72. 10.1016/j.neuroimage.2009.06.060.

15. Power, J.D., Mitra, A., Laumann, T.O., Snyder, A.Z., Schlaggar, B.L., and Petersen, S.E. (2014). Methods to detect, characterize, and remove motion artifact in resting state fMRI. Neuroimage *84*, 320-341. 10.1016/j.neuroimage.2013.08.048.

16. Behzadi, Y., Restom, K., Liau, J., and Liu, T.T. (2007). A component based noise correction method (CompCor) for BOLD and perfusion based fMRI. Neuroimage *37*, 90-101. 10.1016/j.neuroimage.2007.04.042.

17. Satterthwaite, T.D., Elliott, M.A., Gerraty, R.T., Ruparel, K., Loughead, J., Calkins, M.E., Eickhoff, S.B., Hakonarson, H., Gur, R.C., Gur, R.E., and Wolf, D.H. (2013). An improved framework for confound regression and filtering for control of motion artifact in the preprocessing of resting-state functional connectivity data. Neuroimage *64*, 240-256. 10.1016/j.neuroimage.2012.08.052.

18. Patriat, R., Reynolds, R.C., and Birn, R.M. (2017). An improved model of motion-related signal changes in fMRI. Neuroimage *144*, 74-82. 10.1016/j.neuroimage.2016.08.051.

19. Lanczos, C. (1964). Evaluation of Noisy Data. Journal of the Society for Industrial and Applied Mathematics Series B Numerical Analysis *1*, 76-85. 10.1137/0701007.

20. Abraham, A., Pedregosa, F., Eickenberg, M., Gervais, P., Mueller, A., Kossaifi, J., Gramfort, A., Thirion, B., and Varoquaux, G. (2014). Machine learning for neuroimaging with scikit-learn. Frontiers in neuroinformatics *8*, 14. 10.3389/fninf.2014.00014.

21. Jansma, J.M., Ramsey, N.F., Coppola, R., and Kahn, R.S. (2000). Specific versus nonspecific brain activity in a parametric N-back task. Neuroimage *12*, 688-697. 10.1006/nimg.2000.0645.

22. Glasser, M.F., Coalson, T.S., Robinson, E.C., Hacker, C.D., Harwell, J., Yacoub, E., Ugurbil, K., Andersson, J., Beckmann, C.F., Jenkinson, M., et al. (2016). A multi-modal parcellation of human cerebral cortex. Nature *536*, 171-178. 10.1038/nature18933.

23. Brodt, S., Pöhlchen, D., Flanagin, V.L., Glasauer, S., Gais, S., and Schönauer, M. (2016). Rapid and independent memory formation in the parietal cortex. Proceedings of the National Academy of Sciences *113*, 13251-13256. 10.1073/pnas.1605719113.

24. Critchley, H.D., Mathias, C.J., and Dolan, R.J. (2001). Neural activity in the human brain relating to uncertainty and arousal during anticipation. Neuron *29*, 537-545. 10.1016/s0896-6273(01)00225-2.

25. Knutson, K.M., Wood, J.N., and Grafman, J. (2004). Brain activation in processing temporal sequence: an fMRI study. Neuroimage *23*, 1299-1307. 10.1016/j.neuroimage.2004.08.012.

26. Wagner, A.D., Shannon, B.J., Kahn, I., and Buckner, R.L. (2005). Parietal lobe contributions to episodic memory retrieval. Trends Cogn Sci *9*, 445-453. 10.1016/j.tics.2005.07.001.

27. Cai, M.B., Schuck, N.W., Pillow, J.W., and Niv, Y. (2016). A Bayesian method for reducing bias in neural representational similarity analysis. Proceedings of the 30th International Conference on Neural Information Processing Systems. Curran Associates Inc.

28. Mill, R.D., and Cole, M.W. (2023). Neural representation dynamics reveal computational principles of cognitive task learning. bioRxiv. 10.1101/2023.06.27.546751.

29. Liu, Y., Dolan, R.J., Higgins, C., Penagos, H., Woolrich, M.W., Olafsdottir, H.F., Barry, C., Kurth-Nelson, Z., and Behrens, T.E. (2021). Temporally delayed linear modelling (TDLM) measures replay in both animals and humans. Elife *10*. 10.7554/eLife.66917.
