# Supplementary figures and images for: Cost-benefit tradeoff mediates the transition from rule-based to memory-based processing during practice
Source: PLoS Biol. 2025 Jan 23;23(1):e3002987. doi: 10.1371/journal.pbio.3002987 (PMC11793810; doi:10.1371/journal.pbio.3002987)

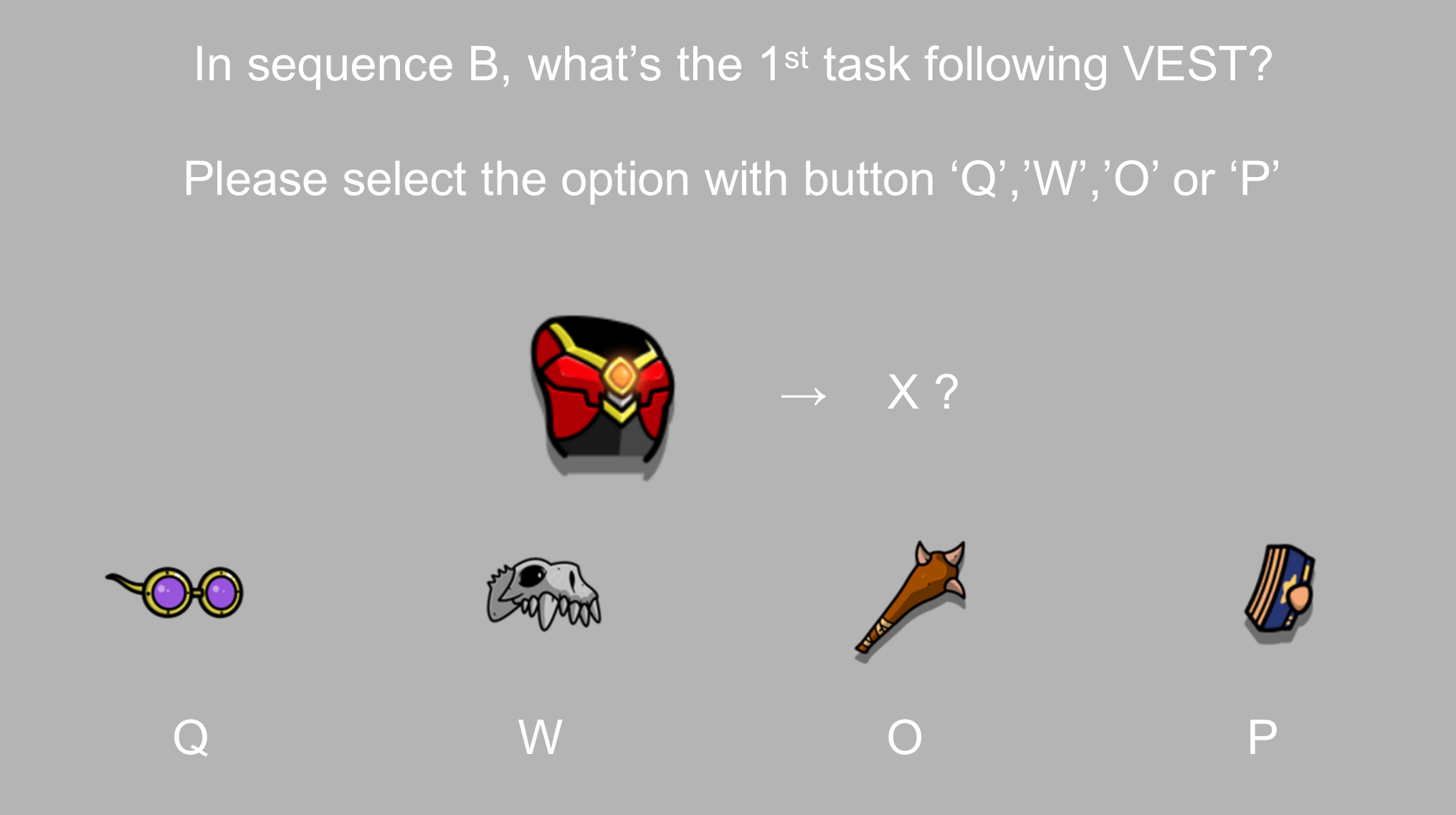

Supplement: S1 Fig — This task aimed to test participants’ memory of task transitions. They were asked to choose the 1st/2nd/3rd tasks (in this example, the 1st task) following a prompted task within one sequence (in this example, sequence “B”). Responses were made with “Q,” “W,” “O,” or “P” buttons on the keyboard without time constraints. (TIF) [file pbio.3002987.s003.tif]

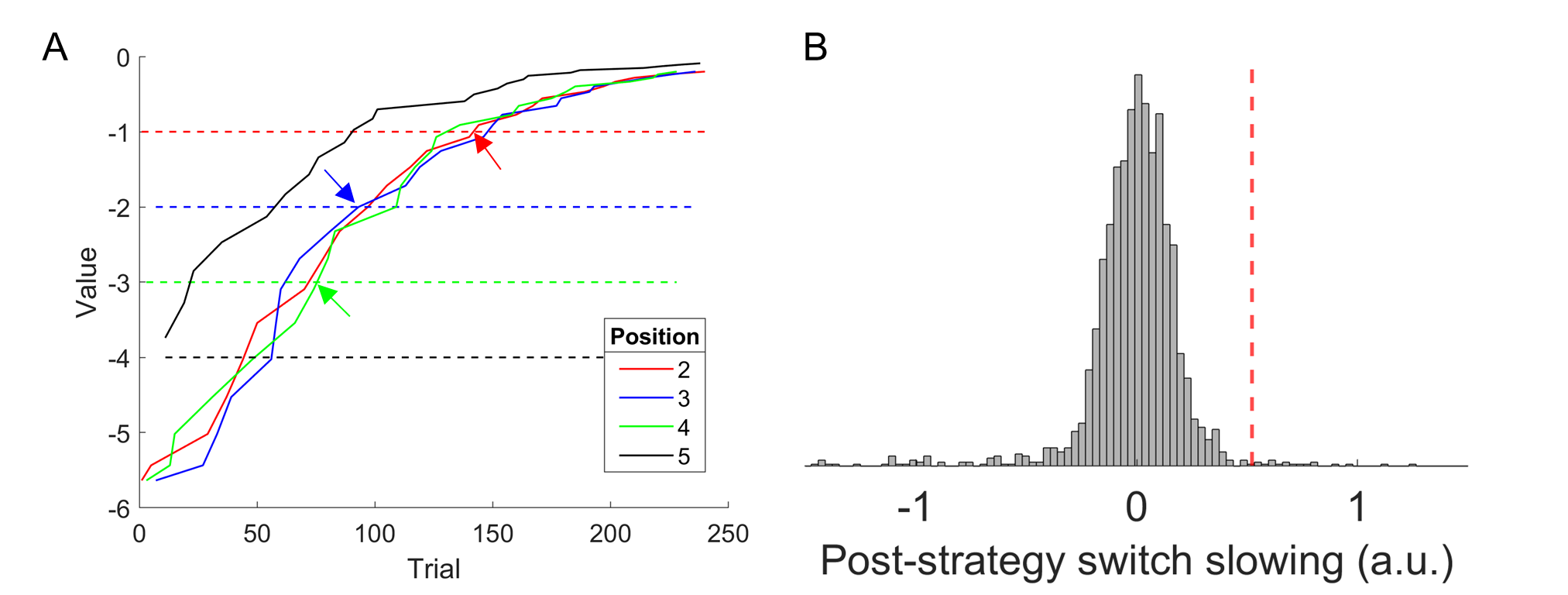

Supplement: S2 Fig — (A) Strategy transition based on the value (i.e., cost-benefit tradeoff) between the rule-based strategy (dashed lines) and the memory strategy (solid lines) in an exemplar sequence of a subject. The arrows indicate the transition points for different cued positions. The value for the rule-based strategy remains constant over time, thus the dashed lines are horizontal. On the other hand, cue-task associations strengthen over learning, thus the solid line increases monotonically. A strategy switch occurs when the 2 lines cross (i.e., when the value of the memory strategy becomes better than that of the rule-based strategy). Note that position 5 has a higher value for memory than for the rule-based strategies throughout all trials, so the participant applied the memory strategy from the beginning. (B) The permutation results (histogram) for the post-strategy switch slowing obtained by randomizing the model parameters. This analysis seeks to examine whether the post-transition slowing is specific to the transition points identified by the model. The red dashed line shows the coefficient derived from real data and is significantly higher than the null distribution, p = 0.005. (TIF) [file pbio.3002987.s004.tif]

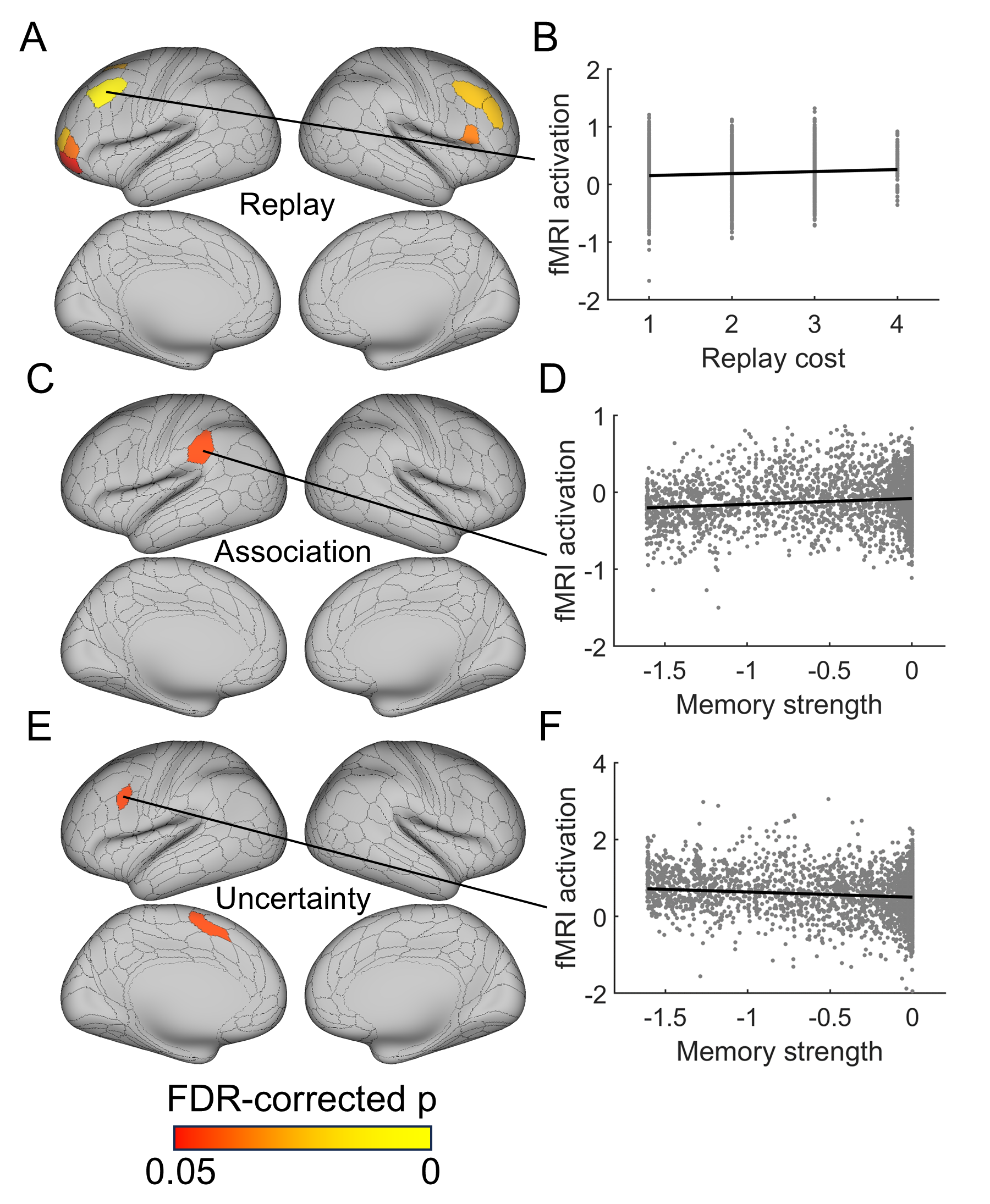

Supplement: S3 Fig — (A) Regions showing higher fMRI activation with higher rule implementation cost (i.e., number of steps replayed). (B) Trial-wise fMRI activation in the left 8C region as a function of rule implementation cost. Data used for (B) can be found in S1 Data, specifically in the sheet labeled “S3B Fig”. (C, E) Show regions with higher trial-wise activation for stronger memory strength (i.e., cue-task associations) and higher trial-wise activation for lower memory strength (i.e., higher uncertainty), respectively. (D, F) Show the trial-wise fMRI activation in the left PF and left IFJp, respectively, plotted as a function of memory strength quantified as reversed entropy. (TIF) [file pbio.3002987.s005.tif]

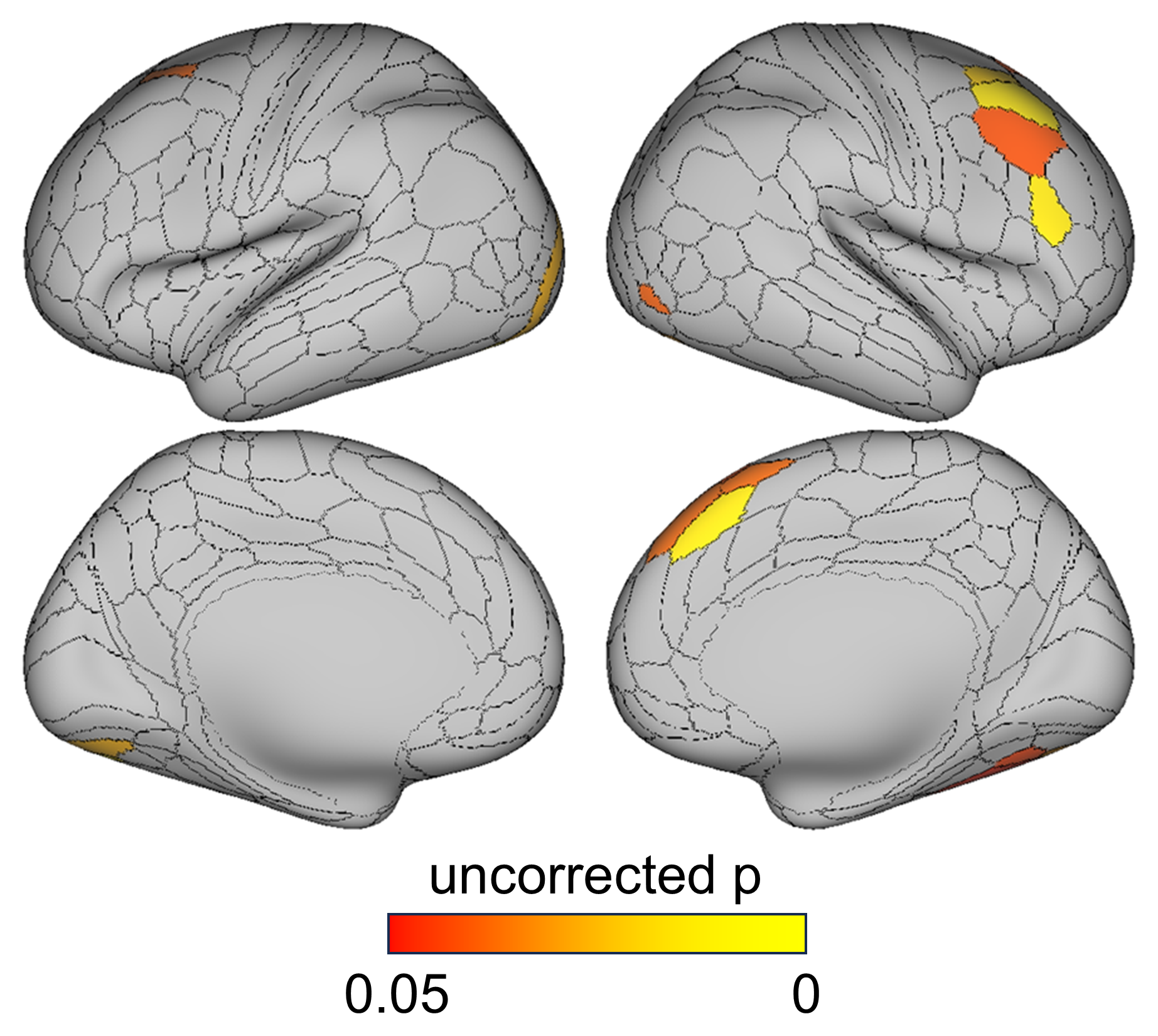

Supplement: S4 Fig — Displayed are regions showing stronger fMRI activation on the first memory trial compared to the last rule trial, thresholded at uncorrected p < 0.05. The involvement of frontal regions is consistent with the switching cost hypothesis that occurs when transferring from one task to another [46,47]. (TIF) [file pbio.3002987.s006.tif]

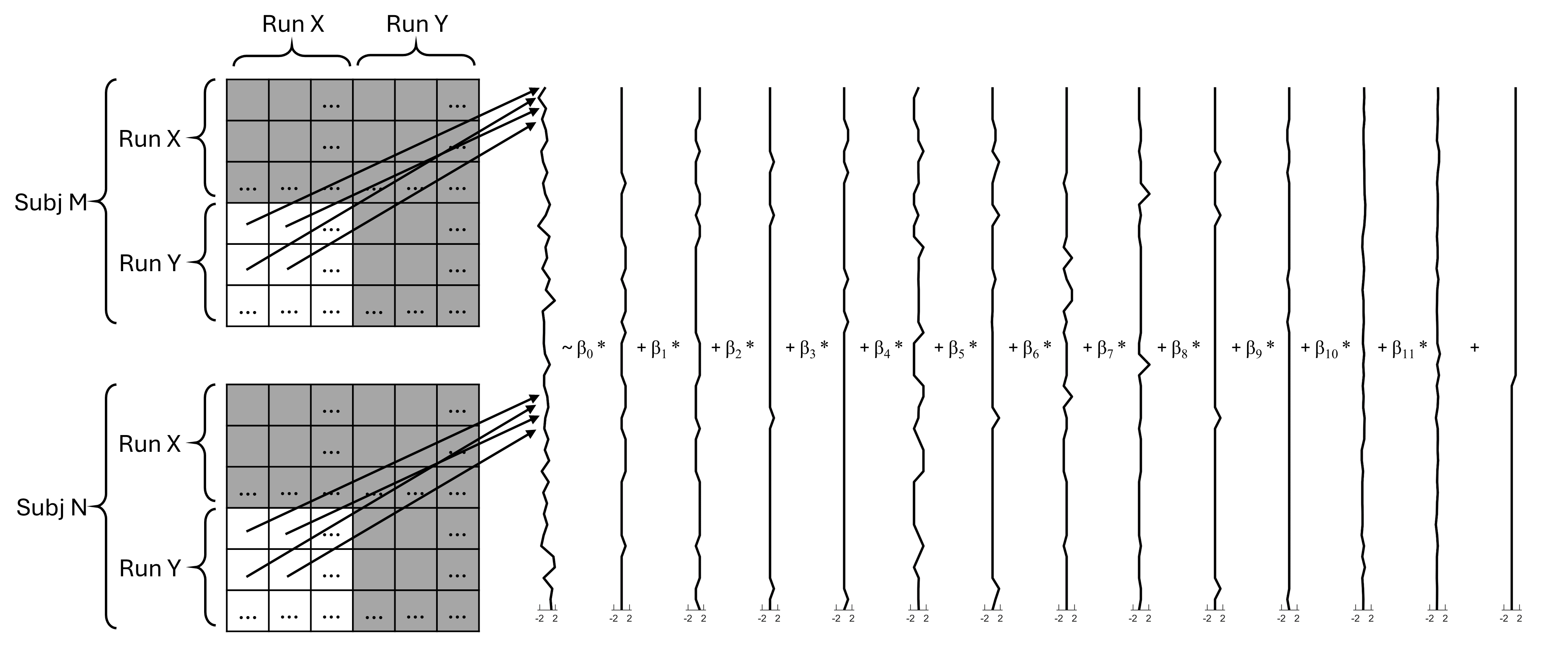

Supplement: S5 Fig — Sample data from 2 participants is plotted to illustrate how the values within the representational similarity matrices are used in the LME. In the left panel, the white cells, representing the similarities/differences in a model variable between 2 trials, are converted into a vector in the LME, while the gray-colored cells are removed due to either within-run correlation (diagonal) or duplication (upright off-diagonal). In the right panel, the first column represents z-transformed representational similarity values. Columns 2 through 13 correspond to regressors for different experimental conditions: R1, R2, M1, M2, RSMRule_R, RSMRule_M, RSMCue_R1, RSMCue_R2, RSMCue_M1, RSM Cue_M2, RSMUnivoxel_trial1 and RSMUnivoxel_trial2. The participant identifier is represented in the final column. (TIF) [file pbio.3002987.s007.tif]

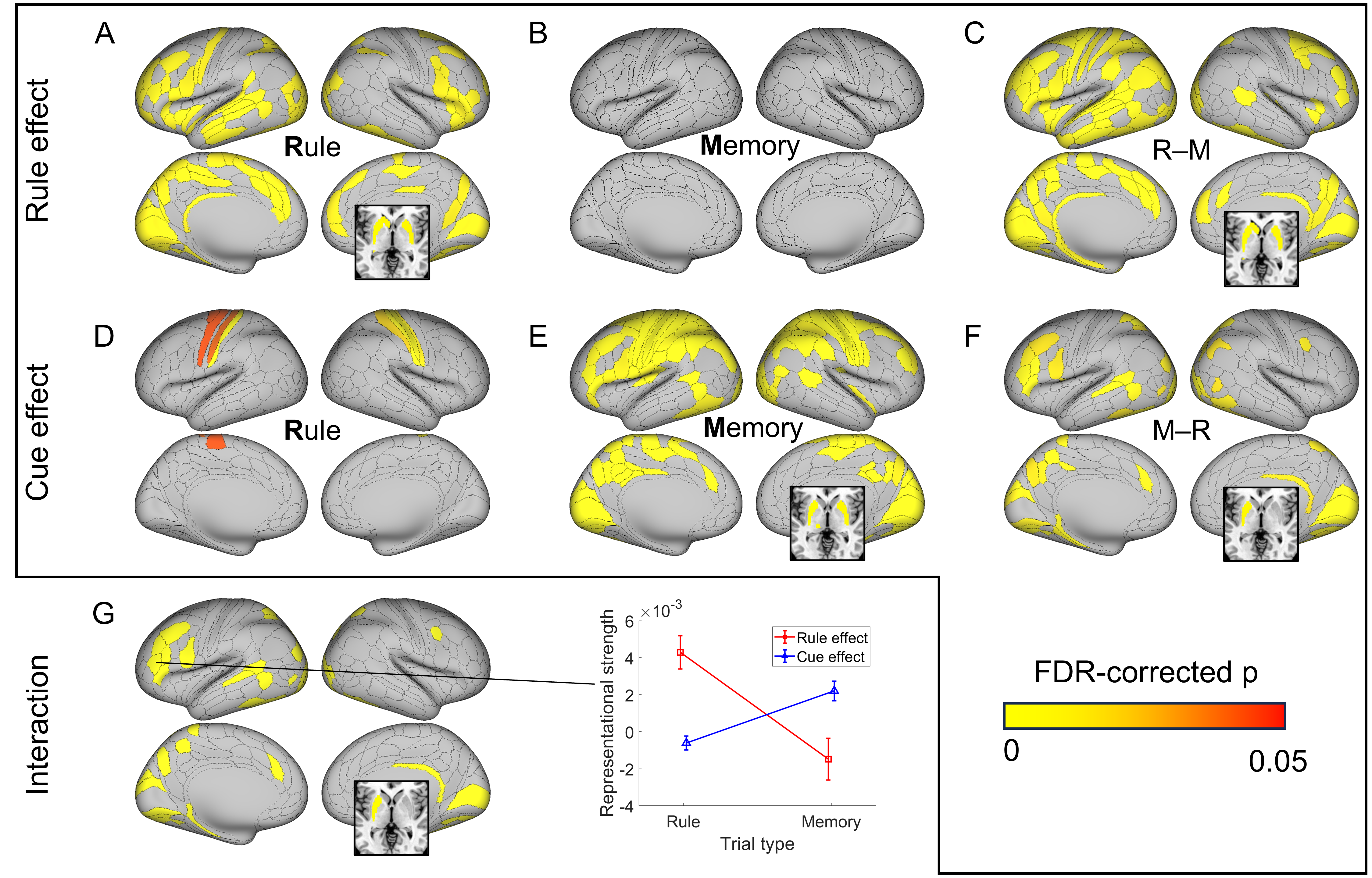

Supplement: S6 Fig — A significant representation of the rule effect is observed on rule (R) trials (panel A) but not on memory (M) trials (panel B). (C) A stronger rule effect on rule than memory trials (R–M) was found in frontoparietal, temporal, occipital, and subcortical regions. In contrast, the cue effect is exhibited in more regions on memory trials (panel E) than on rule trials (panel D), and regions showing a stronger memory effect on memory than rule trials (M–R) include frontoparietal, temporal, occipital, and subcortical regions (panel F). (G) Regions showing double dissociation (i.e., the conjunction of C and F) with both stronger rule effect representation on rule trials and stronger cue effect representation on memory trials. The middle panel illustrates the double dissociation with an example region (left inferior prefrontal sulcus; IFSa). Error bars are standard errors of mean. Data used for (G) can be found in S1 Data, specifically in the sheet labeled “S6G Fig”. All subcortical regions are depicted in an axial slice at z = 0. The right panel illustrates the double dissociation with an example region (left IFSa). (TIF) [file pbio.3002987.s008.tif]

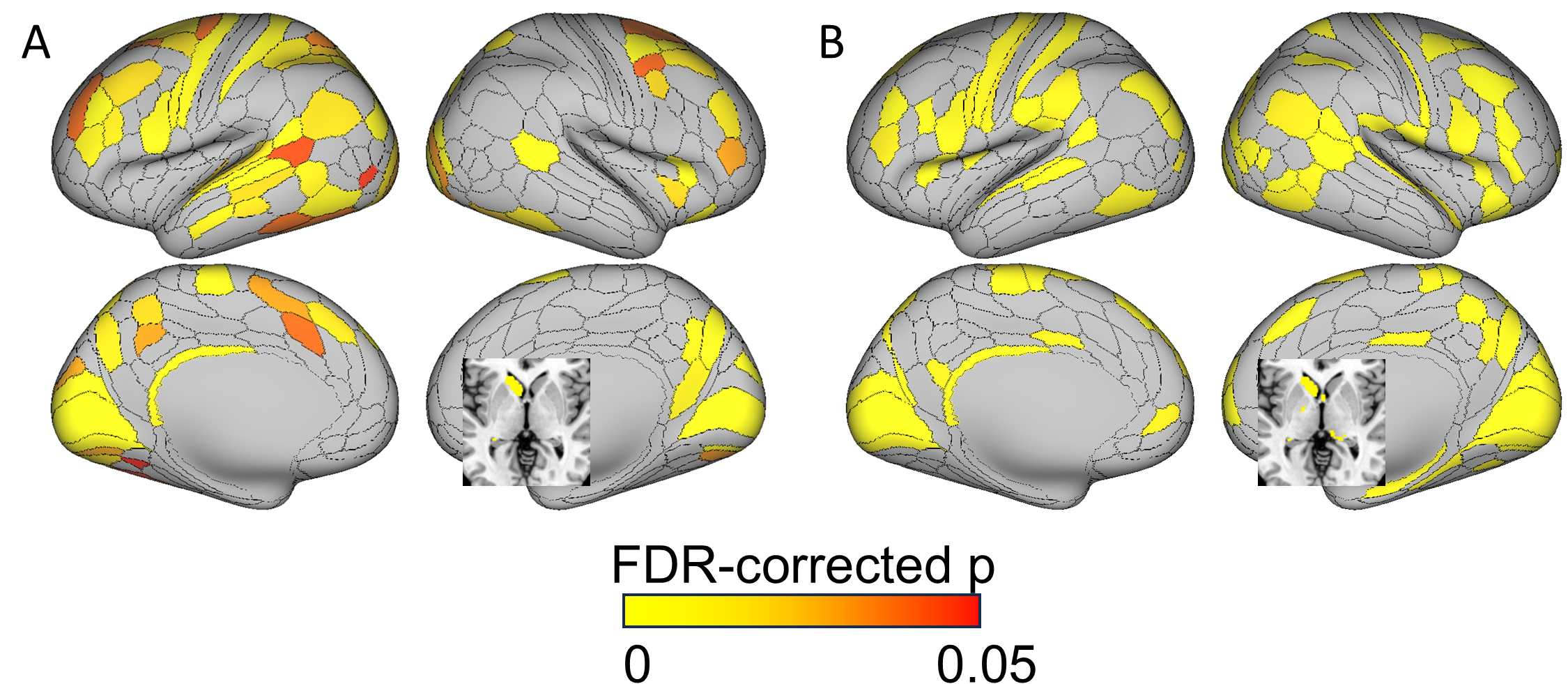

Supplement: S7 Fig — Both panels show regions identified with stronger decoding of replayed tasks on rule than memory trials. (A) FDR-corrected results using regions identified in Fig 4C as search regions. (B) FDR-corrected results using whole-brain ROIs. (TIF) [file pbio.3002987.s009.tif]
