# Supplementary material for: Cost-benefit tradeoff mediates the transition from rule-based to memory-based processing during practice
Source: PLoS Biol. 2025 Jan 23;23(1):e3002987. doi: 10.1371/journal.pbio.3002987 (PMC11793810; doi:10.1371/journal.pbio.3002987)
Supplement: Attachment — Submitted filename: Response to Reviewers.docx [file pbio.3002987.s011.docx]

On behalf of both authors, I have enclosed a revised version of our manuscript. Below, we have provided point-by-point responses to the reviewer’s comments.

The revisions in the manuscript are highlighted in the track changes version. Quoted texts from the manuscript (the clean version) and other literatures are *italicized*.

## Reviewer #1:

In this paper, Yang and Jiang use model comparison and neuroimaging to test a model of how people shift from using rule-based decision making to using memory-based decision making (stimulus-response associations). They find that performance on a task that can be solved using these strategies improves over time. Moreover, model comparison suggests that this happens because people are starting to use the memory-based strategy. Specifically, the model argues that as stimulus-response associations are practiced strategy, their accuracy outweighs the cost of rule-based decision making. The authors contrast these predictions against several other models, and they find that their cost-benefit model provides a better fit to the data. They use neuroimaging and behavioral analyses to argue against the idea that the shift in strategies is driven by a "horse race" model (like Logan's instance theory of automatization). Here, they show that on the first trial where the model predicts a shift towards memory participants seem to incur a switch cost. For example, they show that dlPFC encodes this transition point, and that a broad group of neural regions that encodes the decision variable (the difference between the value for the two strategies).

This is an interesting paper on an old but important question. The computational modeling is sophisticated, and so are the neuroimaging analyses. When I was asked to review this paper, the editor told me my expertise aligned with the (most critical) reviewer #3. I have read their comments, and I agree with the gist behind most of their comments. I also believe that the authors have done a thorough job in responding to them. However, I still have a few remaining concerns. Some of these are more about the structure of the paper, but I also have some theoretical concerns.

**Response:** We would like to thank the reviewer for the positive evaluation of our manuscript and the helpful comments.

My main theoretical concern is that I feel that the horse race model as described by the authors is not a fair representation of the previous reviewer's comments. The authors interpret this model to mean that participants always perform both strategies on each trial. Therefore, when they find that there is no or reduced evidence for "rule" trials later on in the session, they claim that this goes against the prediction of the horse race model. However, I believe that this a fair characterization of a horse race model. First, Logan originally proposed this as a model of automaticity, so the assumption that both algorithms are run in full on each trial seems to be unwarranted. In Logan's (1988) own words (the emphasis is mine):

"When the stimulus is encountered again in the context of the same goal, some proportion of the processing episodes it participated in are retrieved. The subject can then choose to respond on the basis of the retrieved information, if it is coherent and consistent with the goals of the current task, [or to run off the relevant algorithm] and compute an interpretation and a response."

So, even Logan didn't belief that both strategies were active on each trial. Moreover, even if one did believe that both strategies are started, then an easy assumption is that the "losing" one is terminated when the other one wins. Thus, I do not think that the race horse model in this paper (depicted in Figure 1A) is a fair version of the model. I believe that many of the results in this paper that are presented as evidence against the race horse model are consistent with this version of the model. It's not infeasible that on the first trial where the memory strategy wins, there is a switch cost. Moreover, the neuroimaging results that show no evidence for a rule effect on memory trials are perfectly understandable when the memory system wins the race so fast that the rule-based system has no time to seriously get going.

I agree with the previous reviewer #3 that this paper is light on evidence for a cost-benefit decision. The authors argue that each step through the sequence carries some kind of cost. This is not incompatible with the horse race model, where "deeper" sequences just take longer to complete and therefore to win. Based on the reasoning described above, I'm not sure the authors have found compelling evidence for this part of their model.

**Response:** We appreciate the reviewer’s thoughtful comment and agree that our alternative race model alone may not accurately represent Logan’s (1998) original horse-race model. We also acknowledge that the results mentioned in the comment do not directly refute the horse-race model. In the revised manuscript, both our cost-benefit tradeoff model (renamed as the decision-selection model in the revised version) and the original horse-race model are now grounded in Logan’s (1988) work. Specifically, as Logan stated, “*At some point, performance will depend on memory entirely,* ***either as a consequence of statistical properties of the race or because of a strategic decision to trust memory and abandon the algorithm***”, both horse-race and decision-making models should be consistent with the general idea behind Logan’s theory. As the race part of the model has been extensively tested by Logan and colleagues (Compton & Logan, 1991; Logan, 1988), the main aim of our current study is to test whether the strategy transition can be understood within a decision-making framework characterized by a cost-benefit analysis. We believe the decision-making model and the horse-race model together provide full coverage of Logan’s theory. We revised the manuscript throughout to reflect the change of framing. We summarize an overview of the present study in the first paragraph of the Discussion in the revised version (Page 18 in the clean version and Page 20 in the track changes version):

*Practice improves task performance not only by making task execution more efficient but also by changing the strategy used to perform the task. Here, we investigated when and how task strategies and related neural representations change in task learning. After learning two task sequences, participants were asked to report the task type at a cued position from one sequence. The behavioral data showed worse performance at later cued positions, suggesting that participants adopted a strategy to recall (via forward replay) the task sequence to obtain an answer. Additionally, the strength of the cued position effect decreased over time, which could be explained by a transition to a memory-based strategy that retrieves the cued task directly from memory (i.e., performance is not sensitive to cued position). To understand how and when the transition occurs, we incorporated decision-making principles—cost-benefit analysis across strategies and selective strategy implementation—into a decision-selection model. This model accurately predicted transition points, as evidenced by a significant post-transition switch cost (Fig. 2F). Support for the cost-benefit analysis hypothesis also came from the observation that the decision-selection model outperformed a decision-memory-threshold model, which predicts that strategy transition occurs whenever the memory strength of cue-task associations surpasses a certain threshold. We further observed fMRI activation patterns that aligned with a stronger representation of the winning strategy (Fig. 4), consistent with the decision-selection model. Importantly, we observed an encoding of the decision variable (Fig. 5C) that supports the decision-selection model over the both-active model. We then delved into the decision-selection model and found that strategy transition is related to the shift in neural representation in the dlPFC (Fig. 6) and associated with a gradual pattern separation in the vmPFC (Fig. 7).*

We also revised the interpretation of related results as follows:

(1) Regarding the post-transition slowing, we now interpret it as evidence supporting the accurate prediction of strategy transition points, rather than refuting the horse-race model.

(2) We acknowledge that some of our findings could be explained by the horse-race model, as suggested by the reviewer. We added some discussion to clarify this point (Page 19 in the clean version and Page 21 in the track changes version):

*Several brain areas seem to represent both strategies (Fig. 4). This may seem contradictory to the assumption that there are two distinct strategies. We argue that although the two strategies lead to the response in different ways, they indeed share some common cognitive processes, such as visual processing of the cue, memory retrieval and response production. Thus, the same brain regions may be involved in some processes that support both strategies. Crucially, we showed a double dissociation in a subset of these regions, such as the frontoparietal regions (Fig. 4G), where their activation patterns changed with the strategy. This suggests that these brain regions support the two strategies in different ways. The double dissociation results are consistent with the decision-selection model. One alternative explanation to this finding is that the losing strategy was implemented but terminated prematurely as the other strategy winning the race, leading to weaker representations. Thus, the double-dissociation patterns in Fig. 4 are not sufficient to rule out the both-active model. However, we argue that this explanation is not against the conclusion that the winning strategy is represented more strongly than the losing strategy. More importantly, the encoding of the decision variable (Fig. 5C-F) endorses the decision-selection model.*

(3) We agree that the sequence-recalling cost we calculated is consistent with the potential time used in the horse-race model, indicating that our study does not directly challenge the horse-race model. However, this is not the central component of the cost-benefit hypothesis. Instead, we believe the main contribution of the model is to provide a decision-making framework for practice-induced strategy transition. For example, the model can predict the time point of strategy transition, which is consistent with the behavioral finding of post-transition slowing and the stronger neural representation of the winning strategy than the other. To clarify this point, we have revised our conclusion, emphasizing that the decision-making framework offers an alternative explanation for strategy transition without necessarily refuting the race model. It now reads (Page 20 in the clean version and Page 22 in the track changes version):

*In conclusion, the present study demonstrates how and when a transition from a rule-based to a memory-based strategy occurs during practice to improve performance and reduce effort. The strategy transition occurs when the value of a memory-based strategy outweighs that of the rule-based strategy, as determined by a cost-benefit analysis. This process is characterized by a selective representation of the more efficient strategy at any given time, which, while optimizing performance, introduces an event boundary and a cost of transition between different strategy phases. These findings offer a new decision-making framework for understanding how practice is associated with neural and behavioral changes.*

**References**

Compton, B. J., & Logan, G. D. (1991). The transition from algorithm to retrieval in memory-based theories of automaticity. Mem Cognit, 19(2), 151-158. https://doi.org/10.3758/bf03197111

Logan, G. D. (1988). Toward an instance theory of automatization. Psychol Rev, 95(4), 492-527. https://doi.org/10.1037/0033-295x.95.4.492

Finally, it struck me as odd that RT curves in Figure 2A do not actually become flat. As far as I understand, when the memory strategy wins, the order of the cued position should become irrelevant (each cue becomes associated with a response that can be immediately executed). This is particularly true given that the authors report evidence that a deterministic (not stochastic) model provides a superior fit. This suggests that at some point the participants switch to become fully memory based, and therefore these curves should become fully flat by the last block. Why is that not the case?

**Response:** We would like to clarify that our model assumes that the memory strength for position 1 is stronger than 2-4, due to the learned association between each cue and their first task during the training phase. This explains why position 1 consistently shows faster performance than the other positions, contributing to the positive slope.

Our model also assumes equal memory benefits for positions 2, 3, and 4, which predicts a flat pattern for memory trials. The non-flat slopes observed across positions 2 to 4 might be related to the fact that, by the final block, not all participants had fully transitioned to the memory strategy. In fact, only 10 out of 33 participants adopted the memory strategy for all positions 2-4 by block 6. This factor was accounted for by the trial type (termed *Type*) regressor in our regression.

My final major comment is that the paper introduces the task and the modeling philosophy in a way that is too sparse. I'm not intimately familiar with this task and this style of computational modeling, and I needed to pour over the methods and the captions of the figures before I understood the task and modeling rationale. I understand that previous outlet may have been more restrictive in their allotted word count, but I urge the authors to move a lot of the task and modeling details back to the main text.

**Response:** We apologize for any difficulty caused by our prior organization of our manuscript. Following your suggestion, we have reorganized the content to provide more detailed explanations of the relevant methods and rationales in the main text where needed. For example, we have elaborated on the rationales in the section of *Model-free behavioral results* (Page 6 in both the clean version and the track changes version):

*The task involves training and test phases. In the training phase, participants were trained to learn the transition orders of two sequences (A and B) until they remembered transitions between tasks with greater than 90% accuracy (see Methods). In the test phase, they were asked to select the task type of a sequence at a specific position (e.g., A4, indicating the fourth task of sequence A) within the scanner. For the test phase data, we conducted a model-free analysis. Since the rule-based strategy assumes a higher cost for later positions, whereas the memory strategy treats different cued positions equally, a transition from rule-based to memory-based strategy would predict a decreasing cost for later positions over time. To test this hypothesis, we analyzed trial-wise response times (RTs) and error rates (ERs) separately using linear mixed-effects (LME) models.*

Another example is that we added the purposes for including each of the models (M1-M5) in the section *Model-based Behavioral Results* (Page 9 in the clean version and Page 10 in the track changes version):

*We compared M0 to its reduced versions (Table 1, M1-M5) using likelihood ratio tests at the group level. Specifically, M1 assumes behavioral performance doesn’t vary across the two trial types; M2 assumes there is no practice effect of rule implementation; M3 incorporates both assumptions from M1 and M2; M4 assumes only rule-based strategy has been implemented throughout the task; and M5 assumes the model uses neither rule-based nor memory-based strategy.*

In the manuscript, we also provide a conceptual introduction to the key mechanisms of the model in the following manner (Page 3 in both the clean version and the track changes version):

*Conceptually, a memory-based strategy retrieves the answer directly and bypasses the execution of the rules. Thus, it is less effortful and less costly than a rule-based strategy. However, the memory-based strategy depends on the accumulation of experience, meaning its benefit is initially low and gradually increases as the memory becomes stronger with practice. In contrast, the benefit of rule-based processing remains consistently high from the outset. Therefore, early in task learning (e.g., a novice chess player), the rule-based strategy is favored because its higher benefit outweighs its higher cost compared to the memory-based strategy. As more experience is gained (e.g., a veteran chess player), the increased benefit enables the memory-based strategy to outperform the rule-based strategy in the cost-benefit tradeoff, leading to a strategy transition from rule- to memory-based processing. The memory-based strategy remains the winning strategy afterwards.*

Following this comment, we moved the description of the graphical model from the caption of Figure 2 to the main text. To avoid distracting the readers from the main results, we kept the mathematical details of the model in the Methods section.

Finally, and I think this is something that the previous authors also commented on, there are still a lot of grammatical errors in the manuscript, as well as other displays of unconventional English. For example, the first sentence is presented in quotes, but it is not attributed to anyone. There are also several sentences where noun is plural and verb singular, or vice versa ("Strategy switch can occur", "Here, we investigated how task strategy and related neural representations change in task learning"). The authors indicate they have used Grammarly to weed out errors, but I'm afraid that the mere use of this application may not have been sufficient.

**Response:** We apologize for the grammatical errors in the previous version of our manuscript. In this round of revision, we sought the assistance of a native English speaker with two first-author American Psychological Association (APA) journal articles. We hope the revised version meets the language standards for publication.
