# Supplementary material for: Cost-benefit tradeoff mediates the transition from rule-based to memory-based processing during practice
Source: PLoS Biol. 2025 Jan 23;23(1):e3002987. doi: 10.1371/journal.pbio.3002987 (PMC11793810; doi:10.1371/journal.pbio.3002987)
Supplement: Attachment — Submitted filename: Response to Reviewers.docx [file pbio.3002987.s012.docx]

We thank the reviewer for their careful reading and valuable suggestions on the revised manuscript. On behalf of both authors, I have enclosed the latest version of our manuscript. Below, we have provided point-by-point responses to the reviewer’s comments.

The revisions in the manuscript are highlighted in the track changes version. Quoted texts from the manuscript are *italicized*.

## Reviewer #1:

224-226 On each trial, the model performs a cost-benefit analysis to determine which strategy to use based on their values, defined as a weighted sum of _cost and benefit_.
Maybe *their costs and benefits?*

**Response:** We revised this part to “their costs and benefits”.

*On each trial, the model performs a cost-benefit analysis to determine which strategy to use based on their values, defined as a weighted sum of their costs and benefits.*

235. As participants receives feedback
subject verb disagreement

**Response:** We revised this part to :

*As participants receive feedback with practice, the cue-task association becomes stronger.*

254. M4 assumes only rule-based strategy
Missing a?
255-256. M5 assumes the model uses neither rule-based nor memory-based strategy.
Strategies? Or are there two "a"s missing here?

**Response:** We revised this part to:

*M4 assumes only a rule-based strategy has been implemented throughout the task; and M5 assumes the model uses neither rule-based nor memory-based strategies.*

564-566. One alternative explanation to this finding is that the losing strategy was implemented but terminated prematurely as the other strategy winning the race, leading to weaker representations.
"as the other strategy winning the race"

**Response:** We replaced the gerund with the verb format.

*One alternative explanation to this finding is that the losing strategy was implemented but terminated prematurely as the other strategy won the race, leading to weaker representations.*
